# Estimation of life history parameters for river catfish *Eutropiichthys vacha*: insights from multi-models for sustainable management

**DOI:** 10.1016/j.heliyon.2022.e10781

**Published:** 2022-09-29

**Authors:** Dalia Khatun, Md. Yeamin Hossain, Obaidur Rahman, Md. Firose Hossain

**Affiliations:** a Department of Fisheries, University of Rajshahi, Rajshahi 6205, Bangladesh; b Department of Genetic Engineering and Biotechnology, University of Rajshahi, Rajshahi 6205, Bangladesh

**Keywords:** Life history, Size at first sexual maturity, Natural mortality, Optimum catchable length, *Eutropiichthys vacha*, Ganges River

## Abstract

The river catfish, *Eutropiichthys vacha* is a vital protein source for rural communities and has high commercial value, but understanding its life history and management strategies reveals major inadequacies and ambiguities in the riverine ecosystems. Consequently, this study employs multi-models to analyze the life history parameters of *E. vacha* in the Ganges River (northwestern Bangladesh) from January to December, 2020. The total length (TL) and body weight (BW) of 362 individuals (male = 170, female = 192) were measured by a measuring board and a digital weighing balance, respectively. The overall sex ratio (male: female) was 1.0: 1.13 and did not oscillate statistically from the standard 1:1 ratio (*p* > 0.05). The TL varied from 6.7–19.2 cm for males and 6.3–19.0 cm for females. The length-frequency distributions (LFDs) revealed females outnumbered in 8.0–9.99 cm TL whereas males in 7.0–7.99 cm TL. The slope (*b*) of the length-weight relationship (TL *vs.* BW) for both sexes (*b* = 2.87) was substantially lower than isometry, specifying negative allometric growth pattern for *E. vacha*. Sex-specific relative (*K*_*R*_) and Fulton’s (*K*_*F*_) condition analysis revealed better state of well-being of males than females. Only *K*_*F*_ exhibited significant correlation with both BW and TL, hence making it ideal condition for predicting the fitness of *E. vacha* in this river. Moreover, the relative weight (*W*_*R*_) suggests an imbalanced habitat for females with higher abundance of predators but suitable for males. The form factor (*a*_*3.0*_) was 0.0062 and 0.0065, whereas the size at first maturity (*L*_*m*_) and mean natural mortality (*M*_*W*_) were 11.38 and 11.27 cm TL and 1.29 and 1.28 year^−1^ for the respective sexes. Besides, the calculated mean optimum catchable length (*L*_*opt*_) was 13.58 and 13.09 cm TL for each sex. These findings will be crucial for further studies and to recommend appropriate strategy for the sustainable management of *E. vacha* in the Ganges River and adjacent watersheds.

## 1 Introduction

Catfish (order Siluriformes) are the third-largest teleost order after Cypriniformes and Perciformes, with around 4100 species, accounting for about 12% of all teleosts (Eschmeyer and Fong, 2014; Wilson and Reeder, 2005). It gets its name from the whisker-like barbels around their mouths (Talwar and Jhingran, 1991). Catfish have a cosmopolitan distribution and can be found in inland or coastal waterways on all continents, even in Antarctica where fossils have been found (Grande and Eastman, 1986). It is economically significant as a food fish, ornamental fish, and sport fish (Jin et al., 2016). Catfish are a very prominent freshwater fish fauna which is abundantly found in Bangladesh (Rahman, 2005). The immense river systems and inland waters of Bangladesh contain 250 to 266 freshwater fish species (Rahman, 2005; Siddiqui et al., 2007), among which 55 species are classified as catfish (Rahman, 2005), though this number is also stated as 60 in some literature (Sarker et al., 2008). The Batchwa vacha, *Eutropiichthys vacha* (Hamilton, 1822) is a fresh- to brackish- water silurid catfish of the family Schilbeidae under the most diverse order, Siluriformes. It is a pelagic, potamodromous species (Riede, 2004) with voracious feeding habits, mostly feeding on small fishes and insects (Talwar and Jhingran, 1991). This fish is regarded as one of the representative and abundant catfish of the genus *Eutropiichthys* in the Ganges River, although *E. murius* is also available in small quantities (IUCN Bangladesh, 2015). This catfish is generally familiar as Bacha, Kangon, Cherki, and Challi in Bangladesh, India, Nepal, and Pakistan, respectively (Froese and Pauly, 2021). In Bangladesh, it is mostly recognized as a freshwater fish, mostly inhabiting major rivers and their tributaries, *haors*, and *beels* all over the country, but is sometimes also found in coastal rivers and Kaptai Lake (Rahman, 2005; Chowdhury, 2007; Kostori et al., 2011; Bashar et al., 2021). This catfish has a broad spatial distribution that encompasses Bangladesh, Bhutan, India, Myanmar, Nepal, Pakistan, and Thailand (Menon, 1999; Riede, 2004; Talwar and Jhingran, 1991). Due to its excellent flesh quality, it is a highly popular and sought-after consumer food fish (Hasan et al., 2002; Soomro et al., 2007) and has minimal commercial value in the aquarium industry (Abbas, 2010). Moreover, it is a native commercial target fish that is primarily targeted by small-scale and large-scale fishers as a vital source of subsistence (Craig et al., 2004; Hossain et al., 2012). The previous conservational status of *E. vacha* was critically endangered (IUCN Bangladesh, 2000), but currently this species has been assessed as least concern in Bangladesh (IUCN Bangladesh, 2015). However, the wild population is still declining due to over-exploitation, and habitat demolition (IUCN Bangladesh, 2015). Hossain et al. (2017, unpublished) also confirmed the declining trend for this species and mentioned some manmade causes such as overfishing, use of destructive fishing gear (i.e., *Current Jal*) and construction of Farrakka barrage as a major causative factor. Moreover, Khatun et al. (2019) also mentioned how the changing climate may possibly affect the reproduction of *E. vacha* in future in the Ganges River of northwestern Bangladesh. Therefore, this species should be subjected to continuous monitoring for the sustainability in its natural habitat.

The life history features of any fish species in a particular habitat determine its long-term sustainability (Das et al., 2019; Kumar et al., 2014; Prasad et al., 2012). Ample information on life history traits, such as sex ratio and size structure, length–weight relationships, growth, conditions, reproduction, and mortality, is crucial for proper planning and management of an exploited stock (Khatun et al., 2022; Gosavi et al., 2019), particularly when the species is a vital constituent of the commercial fisheries and located at the bottom of the upper food chain (Das et al., 2017; Kumar et al., 2014). Besides, habitat health and the condition of fish can also be delineated from this information (Hossain et al., 2021). Sex ratio (SR) and size structure (length-frequency distribution, LFD) provide the fundamental details for assessing the reproductive potentiality of fish populations (Vazzoler, 1996). The study of LFDs reveals the dynamic correlations between the growth, recruitment, and mortality rate along with breeding phenology, stock status, and habitat condition of riverine fishes (Neuman and Allen, 2001; Ranjan et al., 2005). Length–weight relationships (LWRs) and length–length relationships (LLRs) are important tools in fisheries management because they can distinguish the well-being of fishes belonging to intra- or inter-stock for a specific species (King, 2007). Knowledge of LWRs is fundamental for fisheries management and environmental monitoring schemes in a certain geographical territory (Froese, 2006; Renjithkumar et al., 2021). LWRs are an extensively utilized tool for the estimation of fishery biomass and yield from the length data (Garcia et al., 1998; Froese, 2006; Baitha et al., 2018), and also offer vital information for modeling of aquatic biota (Christensen and Walters, 2004). It can provide insight about the overall condition of a fish species, regarding its growth and survival (Le Cren, 1951; Christensen and Walters, 2004) as well as comparative life histories among diverse topographical localities (Le Cren, 1951; Hossain et al., 2009, 2012; Azad et al., 2018). Besides, LLRs are also essential in fisheries management for relative growth studies where one length type is considered (Hossain et al., 2009; Wang et al., 2015). Moreover, condition factors are regarded as critical tools for assessing the health of fish species as well as the overall aquatic community (Muchlisin et al., 2010) and estimating potential variances among different stocks of identical species (King, 2007). Furthermore, relative weight (*W*_*R*_) is one of the most well-known indexes of fish which can ascertain the prey-predator relationship in a certain water body (Hossain et al., 2021). The size at sexual maturity (*L*_*m*_) is a vital management parameter which can detect the basic reasons for the variation in the maturity size of fishes (Templeman, 1987; Sabbir et al., 2021).

Multi-model inference is a procedure that employs multiple models to estimate parameters instead of just one best model, which has several theoretical and practical benefits (Burnham and Anderson, 2002). Selection of a single model can substantially affect the reliability of inferences, since uncertainty in model selection is often considered to be zero, and thus accuracy is likely overestimated (Katsanevakis, 2006). When more than one model is supported by the data, it might be helpful to model-average the predicted response variable across models in order to draw conclusions that are stronger than those drawn from a single model (Katsanevakis, 2014). In this study, we concentrate on estimating natural mortality (*M*_*W*_) and optimum catchable length (*L*_*opt*_) using multi-models, both of which are vital for population management. The estimation of *M*_*W*_ often represents the current condition of a fish stock and helps to set the management policies accordingly (Brodziak et al., 2011). *L*_*opt*_ is also fundamental in fisheries management policy which specifies the significance of fishing gear selectivity (Mawa et al., 2021).

A good number of studies have been reported on various aspects of *E. vacha* form the Ganges River and other water bodies (Table 1). Although Hossain et al. (2013) reported some parameters of life history including SR, LFD, LWRs, LLRs, condition factors, and form factor (*a*_*3.0*_) of this species from the Jamuna River in northern Bangladesh, the information on *L*_*m*_, *M*_*w*_, and *L*_*opt*_ is lacking. Nonetheless, to the best of the author's knowledge, detailed study on life history utilizing multi-models has not been conducted for this major fishery to date, which is crucial for their proper management and implementation of conservation policy. Therefore, this study is intended to focus on the estimation of life history parameters including sex ratio, length-frequency distributions (LFDs), length-weight relationships (LWRs), conditions (allometric, *K*_*A*_; Fultonʹs, *K*_*F*_; relative, *K*_*R*_; relative weight, *W*_*R*_), form factor (*a*_*3.0*_), size at first sexual maturity (*L*_*m*_), natural mortality (*M*_*W*_), and optimum catchable length (*L*_*opt*_) of *E. vacha* using a number of individuals with various body sizes, which will be useful in management strategy evaluations to support this catfish in the Ganges River in northwestern Bangladesh.

**Table 1:** Available works on different aspects of *Eutropiichthys vacha* (Hamilton, 1822) along with their locations and references.

Aspects	Location/Water body	References
Length-weight and length-length relationship	Ganges River, Bangladesh	Hossain et al. (2009)
	Padma River, Bangladesh	Hossain (2010)
	Atrai and Brahmaputra River, Bangladesh	Islam et al. (2017)
	Betwa and Gomti River, India	Sani et al. (2010)
	Indus River, Pakistan	Soomro et al. (2007)
Sex ratio and size structure	Ganges River, Bangladesh	Khatun et al. (2018)
	Ganga River, India	Tripathi et al. (2015)
Morphometric and meristic	Kaptai Lake, Meghna River and Tanguar *haor*, Bangladesh	Parvej et al. (2014)
Condition factor	Ganges River, Bangladesh	Khatun et al. (2020)
	Atrai and Brahmaputra River, Bangladesh	Islam et al. (2017)
	Ganges River, Bangladesh	Hossain (2010)
Life history traits	Jamuna River, Bangladesh	Hossain et al. (2013)
Sexual maturity, reproduction and feeding habit	Kaptai Lake, Bangladesh	Azadi et al. (1990)
	Ganges River, Bangladesh	Hossain et al*.* (2012)
	Ganges River, Bangladesh	Khatun et al. (2019)
	India	Qasim and Qayyum (1961)
	India	Kar et al. (2006)
	Indus River, Pakistan	Soomro et al*.* (2012)
Population parameters and exploitation status	Kaptai Lake, Bangladesh	Bashar et al. (2021)
	Ganga River, India	Tripathi et al. (2015)
	Indus River, Pakistan	Memon et al. (2017)

## 2 Materials and methods

### 2.1 Study site and fish sampling

**Figure 1:**
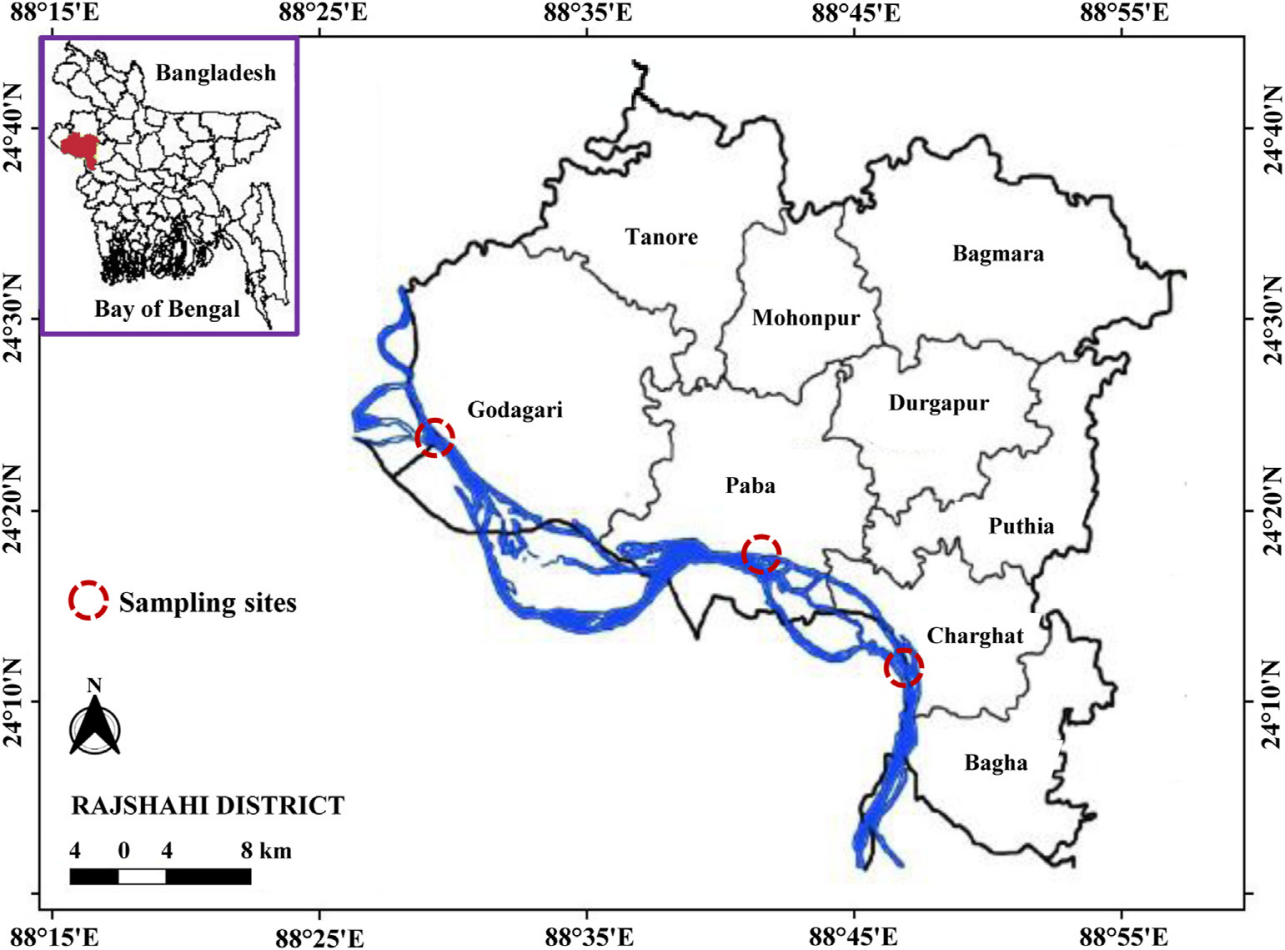
Map showing the study sites in the Ganges River, northwestern Bangladesh. The landing points from where *Eutropiichthys vacha* was collected are indicated by circle.

This study was conducted in the Ganges River (locally renowned as the Padma River in Bangladesh), situated in the northwestern part of Bangladesh. Geographically, this river is located between 24°22′ N latitude, and 88°35′ E longitude (Figure 1). It is well known for having the richest freshwater fish fauna in Bangladesh and serves as a vital feeding and breeding ground for riverine fishes in northwestern Bangladesh (Jones et al., 2003), as well as being home to 26 catfish species (Rahman et al., 2012).

In total, 362 individuals (170 males and 192 females) of *E. vacha* were collected during this study from the various landing points of the Ganges River in the Rajshahi district (Godagari, Paba, and Charghat; Figure 1). All the specimens used in this study originated from the occasional catches of commercial fishers, collected randomly from January to December 2020 using gill net with a mesh size of 1.8–2.2 cm and cast net with 1.5–2.0 cm mesh size. The standard taxonomic identification key given by Talwar and Jhingran (1991) was utilized for the identification of fish species. A random sampling method was followed for the assessment of length and weight to circumvent any prejudice in size. After collection, ice was used to immediately chill the specimens and carried to the laboratory in a cooling box for morphometric analysis.

### 2.2 Fish measurement

Before laboratory examination, all specimens of *E. vacha* were thawed with water. To avoid anomalies in morphometric measures caused by fixation, the morphometric measurements (length and weight) were done on the same day of sampling. The total length (TL), fork length (FL), and standard length (SL) of every specimen were measured by a measuring board, and whole body weight (BW) was recorded using a digital weighing scale with 0.01g precision. For precise weight measurement, specimens were allowed to air dry and wiped with blotting paper to eradicate excess moisture from the surface of the fish. Besides, fish were sexed visually by morphometric differences and microscopically by gonad analysis (Khatun et al., 2019).

### 2.3 Sex ratio (SR) and length-frequency distribution (LFDs)

A chi-square test was used to examine whether there was any disparity in sex ratio from the standard anticipated value of 1:1 (male: female). The length class and the frequency of each length class were assessed according to the estimates of TL. In this study, 1 cm interval of TL was followed during the construction of the length-frequency distribution for both male and female *E. vacha*.

### 2.4 Length-weight and length-length relationships (LWRs and LLRs)

Regression analysis of length-weight data was used to compute the LWRs of *E. vacha* using Le Cren's (1951) power equation as: *BW* = *a×*(TL)^*b*^, where BW is the fish body weight (g), TL is the total length (cm), *a* and *b* is the intercept and slope of the regression, respectively. Linear regression analyses were used to obtain the *a* and *b* parameters based on natural logarithms: ln (*W*) = ln(*a*) + *b* ln (*L*). In addition, the 95% confidence limits of *a* and *b*, as well as the coefficient of determination (*r*^*2*^) were computed according to the regression model's fit (Pervaiz et al., 2012). From the dataset, strong outliers were eliminated after fitting log-log plots of *W* and *L* data via regression analyses (Froese, 2006). To validate whether the growth pattern was isometric or (positive/negative) allometric, a two-tailed t-test was performed to examine the significant divergence from the isometric value of *b* = 3 (Sokal and Rohlf, 1987). In addition, linear regression analysis was used to estimate LLRs such as TL *vs*. FL, TL *vs*. SL, and SL *vs*. FL without log-transformation (Khatun et al., 2018).

### 2.5 Condition factors

The allometric condition factor (*K*_*A*_) was computed by the equation: *K*_*A*_ = *W/L*^*b*^ (Tesch, 1968), where *W* is the BW in g, *L* is the TL in cm, and *b* is the LWRs parameter. Fulton’s condition factor (*K*_*F*_) was determined based on Fulton (1904) equation as: *K*_*F*_ *=100×* (*W/L*^*3*^), where *W* and *L* indicate the same as in *K*_*A*_. To get the *K*_*F*_ closer to unit, a scaling factor of 100 was utilized. Moreover, the equation of Le Cren (1951) was applied for the assessment of the relative condition factor (*K*_*R*_) of *E. vacha* as: *K*_*R*_ = *W/*(*a×L*^*b*^), where *W* and *L* are defined above, and *a* and *b* are LWRs parameters. The relative weight (*W*_*R*_) was calculated from Froese (2006) algorithm as: *W*_*R*_ = (*W/W*_*S*_)*×* 100, where *W* denotes an individual's weight and *W*_*S*_ denotes the expected standard weight for that same individual as computed by *W*_*S*_ = *a×L*^*b*^ (the *a* and *b* variables were retained from the TL *vs.* BW relationship).

### 2.6 Form factor (*a*_*3.0*_) and size at first sexual maturity (*L*_*m*_)

The estimation of *a*_*3.0*_ of *E. vacha* method was done following the Froese (2006) equation as: *a*_*3.0*_ = 10^*log a – s (b−3)*^, where *a* and *b* are described above, and *s* is the slope of regression of *ln a vs. b*. In this study, the *a*_*3.0*_ was estimated using an average slope S = −1.358. Besides, the *L*_*m*_ was estimated by the empirical model given by Binohlan and Froese (2009) as: log (*L*_*m*_) = −0.1189 + 0.9157 × log (*L*_*max*_), where *L*_*max*_ signifies maximum TL.

### 2.7 Natural mortality (*M*_*W*_)

The *M*_*W*_ was computed by the Peterson and Wroblewski (1984) model as *M*_*W*_ = 1.92 year^−1^ ∗(*W*)^−0.25^, where *M*_*W*_ is the natural mortality at body mass *W*, and *W* = *a*×*L*^*b*^, where *L*, *a* and *b* are described earlier. Besides, *M*_*W*_ was also assessed using two other models given by Hoenig (1983) as: exp (1.46–1.01 ln [*L*_*m*_]) and Jensen (1996) as: 1.65/*L*_*m*_ to compare the appropriateness of these three models.

### 2.8 Optimum catchable length (*L*_*opt*_)

The *L*_*opt*_ was assessed using two empirical models to compare the reliability of the estimated value; the first one according to Froese and Binohlan (2000) as: log *L*_*opt*_ = 1.0421×log (*L*_∞_)- 0.2742 and the second one based on the formula of Beverton (1992) as: *L*_*opt*_ = *L*_∞_ {3/(3 + *M/K*)}, where *L*_∞_ is the asymptotic length calculated as: log *L*_∞_ = 0.044 + 0.9841×log (*L*_*max*_) (Froese and Binohlan, 2000), *M* is natural mortality and *K* is the growth co-efficient determined by the equation: *K* = 3/*t*_*max*_ (Pauly and Munro, 1984). The *L*_*opt*_ range was also calculated from the percentage of fish between *L*_*m*_ and *L*_*opt*_ + 10% larger sizes, and the percentage of fish above this *L*_*opt*_ range was designated as mega-spawners (modified from Froese, 2004).

### 2.9 Statistical analyses

GraphPad Prism version 6.5 (GraphPad Software for Windows, San Diego, California, USA) was used to perform statistical analysis on the data. Prior to analysis, the datasets were checked for homogeneity and normality. The comparison of the mean relative weight (*W*_*R*_) with 100 was done using Wilcoxon sign rank test (Anderson and Neumann, 1996). Besides, the correlation between the body measurements (e.g., TL, and BW) with condition factors (*K*_*A*_, *K*_*F*_, *K*_*R*_*,* and *W*_*R*_) was determined by Spearman rank test. Moreover, the LWRs were compared between the sexes by analysis of covariance (ANCOVA). The significance level of all statistical analyses was determined to be 5% (*p* < 0.05).

## 3 Results

### 3.1 Sex ratio (SR) and length-frequency distributions (LFDs)

In this study, 47% of the 362 sampled individuals of *E. vacha* were males and 53% were females, with the total sex ratio not differing significantly from the typical 1:1 ratio (*df* = 1, *χ*^2^ = 1.34*, p* > 0.05) (Table 2). However, the length class based distinction in sex ratio revealed a dominance of females in the 8.00–9.99 cm TL size groups, while males in the 7.00–7.99 cm TL range, although no statistically substantial differences (*p* > 0.05) was observed.

**Table 2:** Number of males, females, and sex ratio (male: female = 1:1) of *Eutropiichthys vacha* (Hamilton, 1822) from the Ganges River, northwestern Bangladesh.

Length class (TL, cm)	Number of specimens			Sex ratio	*χ*^2^ (*df* = 1)	Significance
	Male	Female	Total	(Male/Female)		
6.00–6.99	2	4	6	1 : 2.00	0.67	*ns*
7.00–7.99	16	6	22	1 : 0.38	4.55	*∗*
8.00–8.99	32	39	71	1 : 1.22	0.69	*ns*
9.00–9.99	39	55	94	1 : 1.41	2.72	*ns*
10.00–10.99	20	32	52	1 : 1.60	2.77	*ns*
11.00–11.99	6	6	12	1 : 1.00	0.00	*ns*
12.00–12.99	10	8	18	1 : 0.80	0.22	*ns*
13.00–13.99	6	1	7	1 : 0.17	3.57	*ns*
14.00–14.99	7	8	15	1 : 1.14	0.07	*ns*
15.00–15.99	8	7	15	1 : 0.88	0.07	*ns*
16.00–16.99	7	8	15	1 : 1.14	0.07	*ns*
17.00–17.99	9	9	18	1 : 1.00	0.00	*ns*
18.00–18.99	6	8	14	1 : 1.33	0.29	*ns*
19.00–19.99	2	1	3	1 : 0.50	0.33	*ns*
**Overall**	**170**	**192**	**362**	1 : 1.13	**1.34**	***ns***

TL, total length; *df*, degree of freedom; *ns*, not significant; ∗, significant at 5% level (*χ*^2^_t 1, 0.05_ = 3.84).

**Figure 2:**
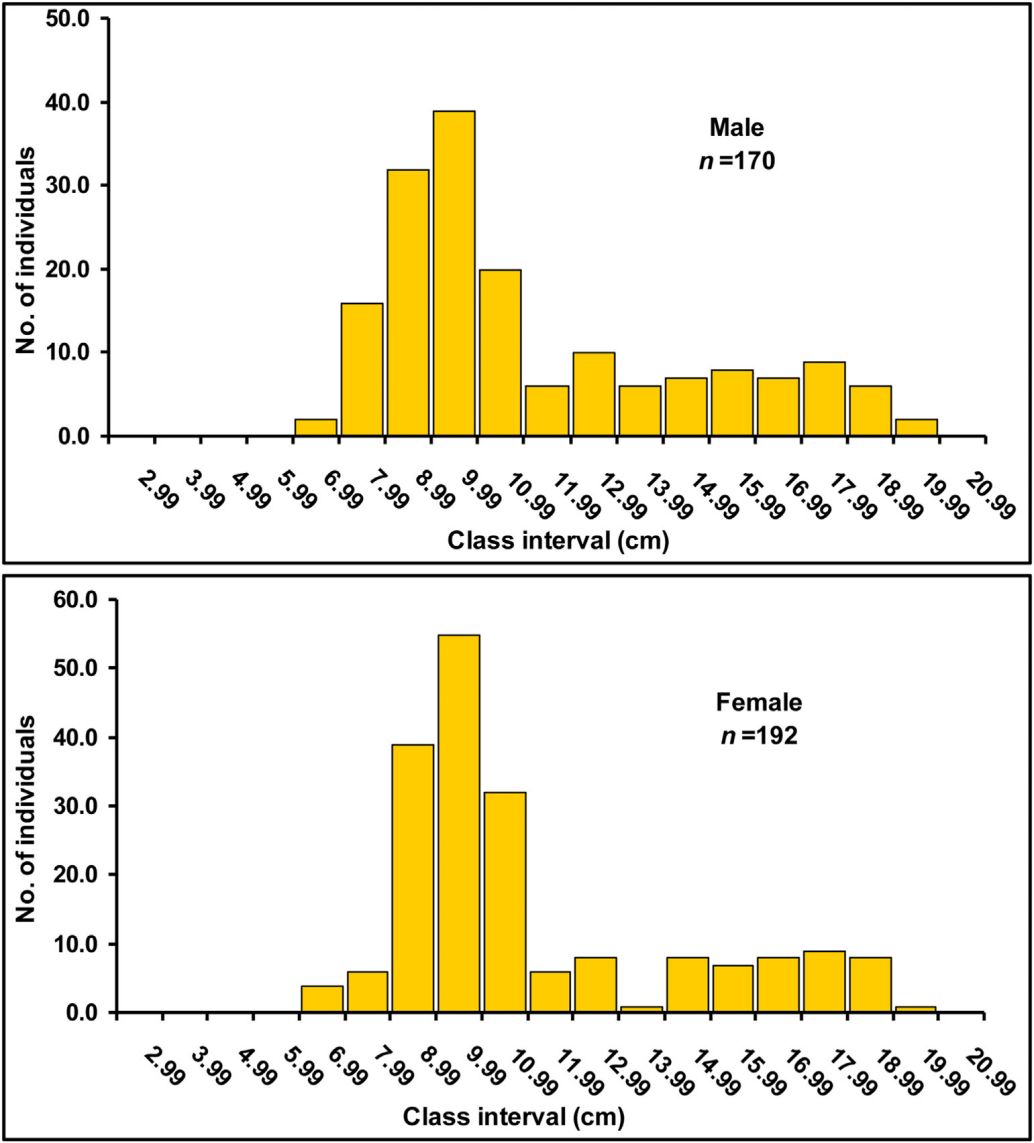
The length-frequency distribution of male and female *Eutropiichthys vacha* (Hamilton, 1822) in the Ganges River, northwestern Bangladesh.

**Figure 3:**
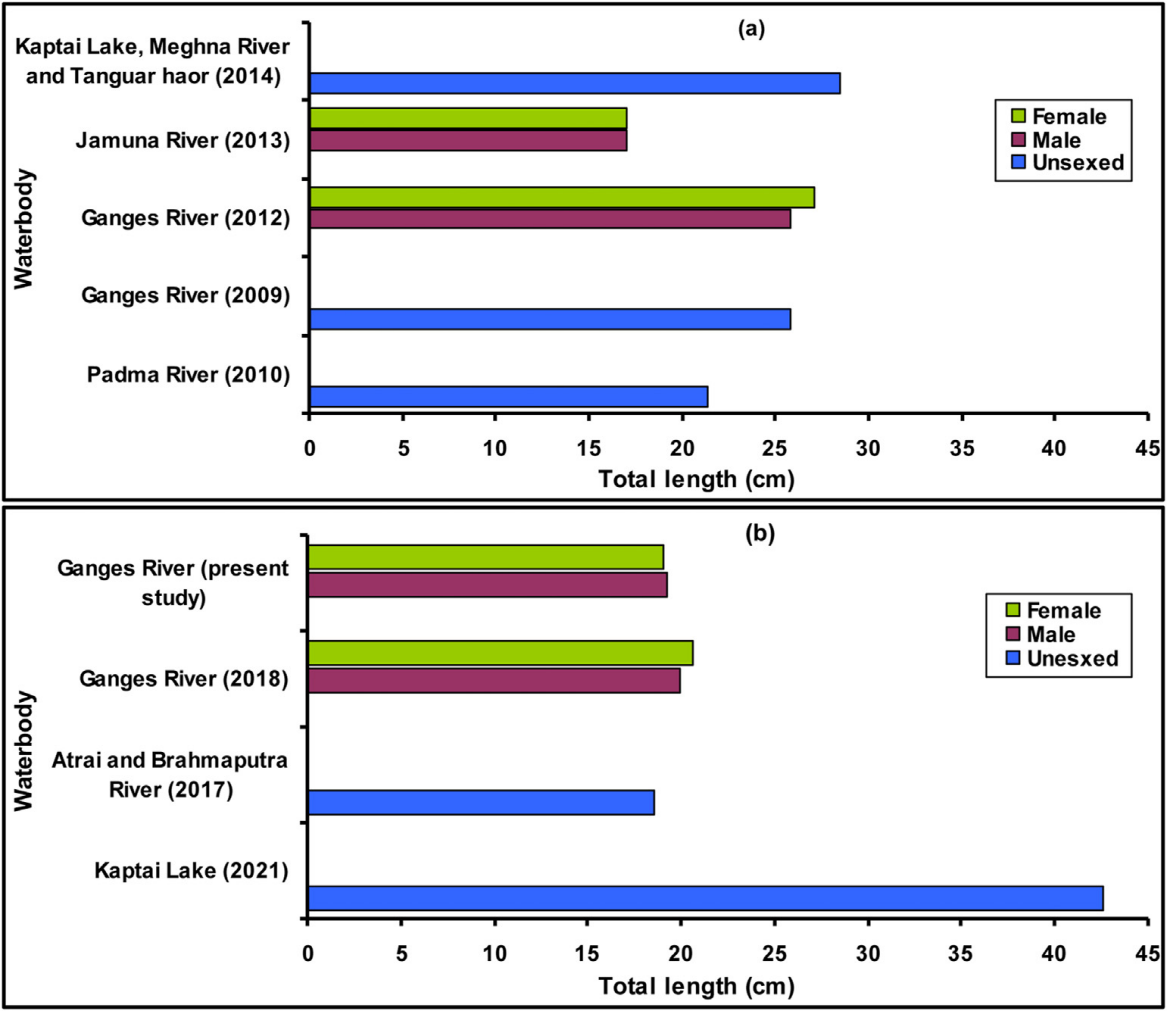
Total length of *Eutropiichthys vacha* (Hamilton, 1822) collected from various waterbodies of Bangladesh during (a) Pre-IUCN Bangladesh (2015) and (b) Post-IUCN Bangladesh (2015) assessments. Sampling year are given in parenthesis.

Table 3 demonstrates the descriptive statistics of length and weight measurements of *E. vacha* as well as their 95% confidence limit (CL). The smallest and largest individuals measured in this study were 6.3 cm and 19.2 cm TL, respectively, with BW spanning between 1.80 to 45.65 g, irrespective of sex. According to LFDs, the 8.00–9.99 cm TL size group was proportionately dominating for both males and females (representing 42% and 49% of the total population, respectively) in the Ganges River (Figure 2). The data of TL and BW of both sexes did not passed the normality test and thus non-parametric Mann-Whitney U-Test was used for comparison between sexes. LFD revealed no significant difference in TL between sexes (U = 16106, *p* = 0.830), and BW also exhibited similar findings (U = 15639, *p* = 0.494). Figure 3 represents the changes of TL of *E. vacha* from the available literatures of Bangladesh during pre and post IUCN Bangladesh (2015) assessment.

**Table 3:** Descriptive statistics of length and weight measurements of *Eutropiichthys vacha* (Hamilton, 1822) specimens in the Ganges River, Bangladesh.

Characteristics	*n*	Min	Max	Mean ± SD	95% CL
**Male**					
Total length (cm)	170	6.7	19.2	11.167 ± 3.331	10.663–11.671
Fork length (cm)		5.9	16.6	9.806 ± 2.879	9.371–10.242
Standard length (cm)		5.3	15.2	8.902 ± 2.672	8.489–9.307
Body weight (g)		1.8	43.87	11.992 ± 10.911	10.340–0.606
**Female**					
Total length (cm)	192	6.3	19.0	11.021 ± 3.151	10.573–11.470
Fork length (cm)		5.5	16.6	9.678 ± 2.694	9.295–10.062
Standard length (cm)		4.9	15.1	8.777 ± 2.448	8.429–9.126
Body weight (g)		1.86	45.65	11.913 ± 10.963	10.353–13.474
**Combined**					
Total length (cm)	362	6.3	19.2	11.090 ± 3.233	10.756–11.424
Fork length (cm)		5.5	16.6	9.738 ± 3.779	9.451–10.026
Standard length (cm)		4.9	15.2	8.836 ± 2.553	8.572–9.100
Body weight (g)		1.8	45.65	11.950 ± 10.923	10.821–13.079

*n*, sample size; Min, minimum; Max, maximum; SD, standard deviation; CL, confidence limit.

### 3.2 Length-weight and length-length relationships (LWRs and LLRs)

**Figure 4:**
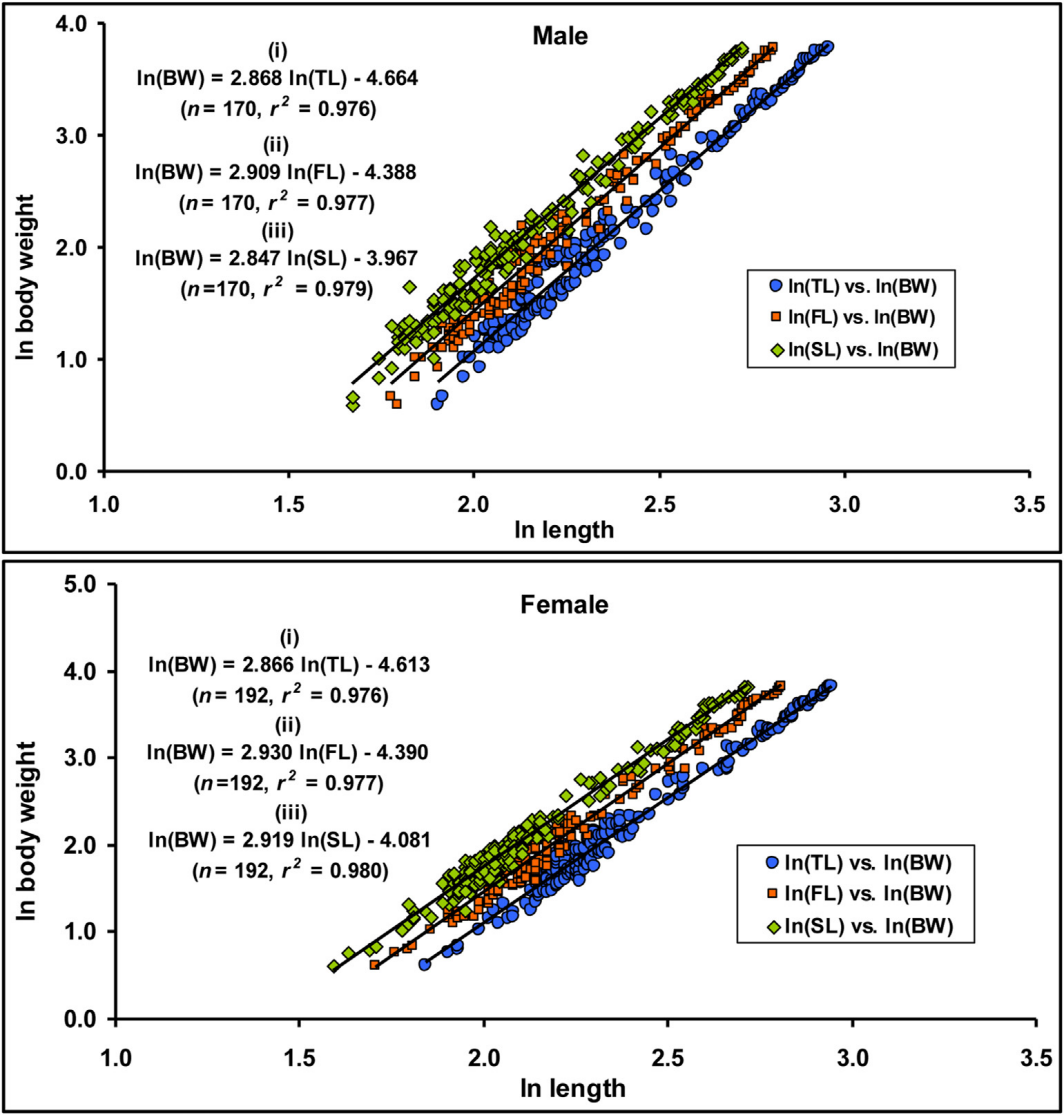
Relationships (*ln W* = *ln a + b lnL*) between (i) ln total length *vs.* ln body weight, (ii) ln fork length *vs.* ln body weight and (iii) ln standard length *vs.* ln body weight of male and female *Eutropiichthys vacha* (Hamilton, 1822) in the Ganges River, northwestern Bangladesh.

Table 4 and Figure 4 represents the findings of the LWRs of *E. vacha*, including sample sizes (*n*), values for regression parameters (*a* and *b*) of LWRs, as well as their corresponding 95% confidence limits, coefficients of correlation (*r*^*2*^), and growth type. The estimated *b* values derived from the LWRs were significantly lower than 3.0 (*b* < 3.00, *p* < 0.001), specifying negative allometric growth (A-) for both males and females, as well as combined sexes. This denotes that the length grows faster than the weight for this species in the Ganges River. Statistically, a highly significant correlation (*p* < 0.001) was observed for all LWRs with the *r*^*2*^ values ≥ 0.975. Also, significant sex differences in LWRs were revealed by ANCOVA (*p* = 0.012) for the studied species.

**Table 4:** Descriptive statistics and estimated parameters of the length-weight relationships (BW = *a*×*L*^*b*^) of *Eutropiichthys vacha* (Hamilton, 1822) from the Ganges River, northwestern Bangladesh.

Equation	Sex	*n*	Regression parameters		95% CL of *a*	95% CL of *b*	*r*^*2*^	GT
			*a*	*b*				
BW = *a*×TL^*b*^	M	170	0.0094	2.87	0.0080–0.0111	2.798–2.937	0.976	–A
BW = *a*×FL^*b*^			0.0124	2.91	0.0107–0.0145	2.841–2.977	0.977	
BW = *a*×SL^*b*^			0.0189	2.85	0.0165–0.0217	2.784–2.909	0.980	
BW = *a*×TL^*b*^	F	192	0.0099	2.87	0.0085–0.0116	2.801–2.930	0.976	–A
BW = *a*×FL^*b*^			0.0124	2.93	0.0107–0.0143	2.866–2.994	0.977	
BW = *a*×SL^*b*^			0.0169	2.92	0.0149–0.0192	2.860–2.979	0.980	
BW = *a*×TL^*b*^	C	362	0.0097	2.87	0.0087–0.0109	2.817–2.913	0.975	–A
BW = *a*×FL^*b*^			0.0125	2.92	0.0112–0.0139	2.871–2.965	0.976	
BW = *a*×SL^*b*^			0.0180	2.88	0.0164–0.0198	2.835–2.925	0.979	

BW, body weight; TL, total length; FL, fork length; SL, standard length; M, male; F, female; C, combined sex; *n*, sample size; *a*, intercept; *b*, slope; CL, confidence limit for mean values; *r*^*2*^, coefficient of determination; GT, growth type; –A, negative allometric.

Furthermore, Table 5 depicts the LLRs (TL *vs.* SL, TL *vs.* FL, and SL *vs.* FL), together with the calculated parameters (*a* and *b*) and their respective 95% CL, and the coefficient of determination (*r*^*2*^). In this study, all LLRs were highly significant (*p* < 0.001), with the *r*^*2*^ value exceeding 0.995.

**Table 5:** The estimated parameters of the length-length relationships (*y = a +b × x*) of *Eutropiichthys vacha* (Hamilton, 1822) from the Ganges River, northwestern Bangladesh.

Equation	Sex	Regression parameters		95% CL of *a*	95% CL of *b*	*r*^*2*^
		*a*	*b*			
TL = *a* + *b ×* FL	M	–0.1686	1.1559	–0.2468 to –0.0904	1.1483–1.1636	0.998
TL = *a* + *b ×* SL		0.0973	1.2435	–0.0306 to 0.2252	1.2297–1.2572	0.995
SL = *a* + *b ×* FL		–0.1776	0.9259	–0.2752 to –0.0799	0.9164–0.9355	0.995
TL = *a* + *b ×* FL	F	–0.2880	1.1685	–0.3702 to –0.2058	1.1603–1.1767	0.998
TL = *a* + *b ×* SL		0.2546	1.2847	–0.3560 to –0.1531	1.2736–1.2958	0.996
SL = *a* + *b ×* FL		–0.0072	0.9076	–0.0790 to –0.0646	0.9005–0.9148	0.997
TL = *a* + *b ×* FL	C	–0.2281	1.1622	–0.2850 to –0.1712	1.1566–1.1678	0.998
TL = *a* + *b ×* SL		–0.0742	1.2635	–0.1571 to 0.0087	1.2545–1.2725	0.995
SL = *a* + *b ×* FL		–0.0928	0.9169	–0.1532 to –0.0325	0.9109–0.9228	0.996

TL, total length; FL, fork length; SL, standard length; M, male; F, female; C, combined sex; *a*, intercept; *b*, slope; CL, confidence limit for mean values; *r*^*2*^, coefficient of determination.

### 3.3 Condition factors

**Figure 5:**
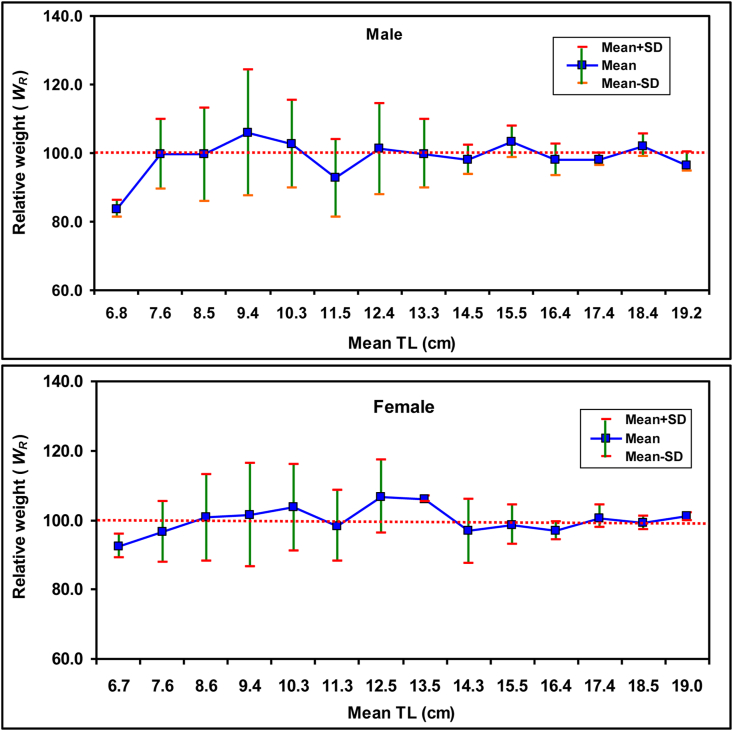
The relationship between total length and relative weight of male and female *Eutropiichthys vacha* (Hamilton, 1822) in the Ganges River, northwestern Bangladesh.

Normality of all four studied condition factors between sexes were tested. However none of them were normally distributed, hence non-parametric Mann-Whitney U-test were employed to find significant differences between the sexes. The *K*_*A*_ values varied from 0.0073 – 0.0141 for males and 0.0066–0.0112 for females (Table 6), and Mann-Whitney U-test showed significant differences of *K*_*A*_ between the sexes (*p* < 0.0001). The *K*_*F*_ ranged from 0.5253 – 1.0439 for males and 0.5527–0.9413 for females and the calculated range of *K*_*R*_ was 0.7747–1.4961 and 0.3920–0.6654 for both sexes, respectively (Table 6). Similar to *K*_*A*_, Mann-Whitney U-test stated significant distinction between males and females (*p* < 0.0001) for both *K*_*F*_ and *K*_*R*_. The calculated *W*_*R*_ for males was 77.4737–149.6149 and for females it was 75.679–128.155 (Table 6). The relationships of condition factors (*K*_*A*_*, K*_*F*_*, K*_*R*_*,* and *W*_*R*_) with TL and BW which are given in Table 7, were also non-normally distributed, consequently non-parametric spearman rank (*r*_*s*_) test was employed. Among the calculated four condition factors in this study, only *K*_*F*_ showed significant correlation with both TL (*r*_*s*_ = −0.2040, *p* = 0.0076 for males and *r*_*s*_ = –0.2166, *p* = 0.0026 for females) and BW ((*r*_*s*_ = −0.2409, *p* = 0.0026 for males and *r*_*s*_ = −0.3204, *p* < 0.001 for females). Figure 5 illustrates the relationship between TL and *W*_*R*_ of *E. vacha*. The *W*_*R*_ of females in this study showed statistically significant deviations from 100 (*p* < 0.0001), whereas males showed an insignificant deviation (*p* = 0.8447), as demonstrated by the non-parametric Wilcoxon sign rank test due to the failure of the dataset to pass the normality test.

**Table 6:** Allometric (*K*_*A*_), Fulton′s (*K*_*F*_), and relative condition factors (*K*_*R*_) and relative weight (*W*_*R*_) of *Eutropiichthys vacha* (Hamilton, 1822) from the Ganges River, northwestern Bangladesh.

Condition factors	Sex	*n*	Min	Max	Mean ± SD	95% CL
*K*_*A*_	M	170	0.0073	0.0141	0.0095 ± 0.0012	0.0093–0.0097
*K*_*F*_			0.5253	1.0439	0.6944 ± 0.0950	0.6801–0.7088
*K*_*R*_			0.7747	1.4961	1.0107 ± 0.1305	0.9910–1.0305
*W*_*R*_			77.4737	149.6149	101.0725 ± 13.0476	99.0970–103.0480
*K*_*A*_	F	192	0.0066	0.0112	0.0088 ± 0.0010	0.0086–0.0089
*K*_*F*_			0.5527	0.9413	0.7272 ± 0.0902	0.7134–0.7400
*K*_*R*_			0.3920	0.6654	0.5197 ± 0.0618	0.5109–0.5285
*W*_*R*_			75.6790	128.1550	100.8826 ± 11.8304	99.1986–102.5667
*K*_*A*_	C	362	0.0073	0.0141	0.0098 ± 0.0012	0.0097–0.0099
*K*_*F*_			0.5252	1.0439	0.7118 ± 0.0938	0.7021–0.7215
*K*_*R*_			0.7552	1.4577	1.0091 ± 0.1257	0.9961–1.0221
*W*_*R*_			75.5236	145.7732	100.9084 ± 12.5698	99.6092–102.2076

*K*_*A*_, allometric condition factor; *K*_*F*,_ Fulton′s condition factor; *K*_*R*,_ relative condition factor; *W*_*R*_, relative weight; M, male; F, female; C, combined sex; *n*, sample size; Min, minimum; Max, maximum; SD, standard deviation; CL, confidence limit for mean values.

**Table 7:** Relationship of condition factors with total length (TL) and body weight (BW) of *Eutropiichthys vacha* (Hamilton, 1822) from the Ganges River, northwestern Bangladesh.

Relationships	Sex	*r*_*s*_ values	95% CL of *r*_*s*_	*p* values	Significance
TL *vs. K*_*A*_	M	0.0390	‒0.1166 to 0.1928	0.6135	*ns*
TL *vs. K*_*F*_		‒0.2040	‒0.3480 to ‒0.0507	0.0076	*∗∗*
TL *vs. K*_*R*_		0.0704	‒0.0855 to 0.2229	0.3617	*ns*
TL *vs. W*_*R*_		0.0708	‒0.0851 to 0.2233	0.3588	*ns*
BW *vs. K*_*A*_		0.2316	0.0795 to 0.3732	0.0024	∗∗
BW *vs. K*_*F*_		‒0.2409	‒0.4697 to 0.0801	0.0026	*∗∗*
BW *vs. K*_*R*_		0.2679	0.1178 to 0.4060	0.0004	∗∗∗
BW *vs. W*_*R*_		0.2682	0.1182 to 0.4063	0.0004	∗∗∗
TL *vs. K*_*A*_	F	‒0.1091	‒0.2508 to 0.0373	0.1321	*ns*
TL *vs. K*_*F*_		‒0.2166	‒0.3512 to ‒0.0731	0.0026	∗∗
TL *vs. K*_*R*_		‒0.0449	‒0.1894 to 0.1015	0.5363	*ns*
TL *vs. W*_*R*_		‒0.0446	‒0.1892 to 0.1018	0.5388	*ns*
BW *vs. K*_*A*_		0.1391	‒0.0069 to 0.2792	0.0544	*ns*
BW *vs. K*_*F*_		‒0.3204	‒0.4145 to ‒0.2767	<0.0001	∗∗∗
BW *vs. K*_*R*_		0.2118	0.0682 to 0.3469	0.0032	∗∗
BW *vs. W*_*R*_		0.2121	0.0685 to 0.3472	0.0031	∗∗
TL *vs. K*_*A*_	C	0.0245	‒0.0818 to 0.1303	0.6420	*ns*
TL *vs. K*_*F*_		‒0.2141	‒0.3132 to ‒0.1105	<0.0001	∗∗∗∗
TL *vs. K*_*R*_		0.0581	‒0.0483 to 0.1633	0.2700	*ns*
TL *vs. W*_*R*_		0.0586	‒0.0478 to 0.1637	0.2660	*ns*
BW *vs. K*_*A*_		0.2485	0.1462 to 0.3455	<0.0001	∗∗∗∗
BW *vs. K*_*F*_		‒0.3072	‒0.4989 to ‒0.1133	0.0054	∗∗
BW *vs. K*_*R*_		0.2885	0.1881 to 0.3829	<0.0001	∗∗∗∗
BW *vs. W*_*R*_		0.2890	0.1886 to 0.3833	<0.0001	∗∗∗∗

TL, total length; BW, body weight; *K*_*A*_, allometric condition factor; *K*_*F*_; Fulton′s condition factor; *K*_*R*_, relative condition factor; *W*_*R*_, relative weight; M, male; F, female; C, combined sex; *r*_*s*_, Spearman rank-correlation values; CL, confidence limit; *p*, shows the level of significance; *ns*, not significant; ∗ significant (*p* ≤ 0.005); ∗∗ highly significant (*p* ≤ 0.01); ∗∗∗ very highly significant (*p* ≤ 0.001).

### 3.4 Form factor (*a*_*3.0*_) and size at fist sexual maturity (*L*_*m*_)

**Figure 6:**
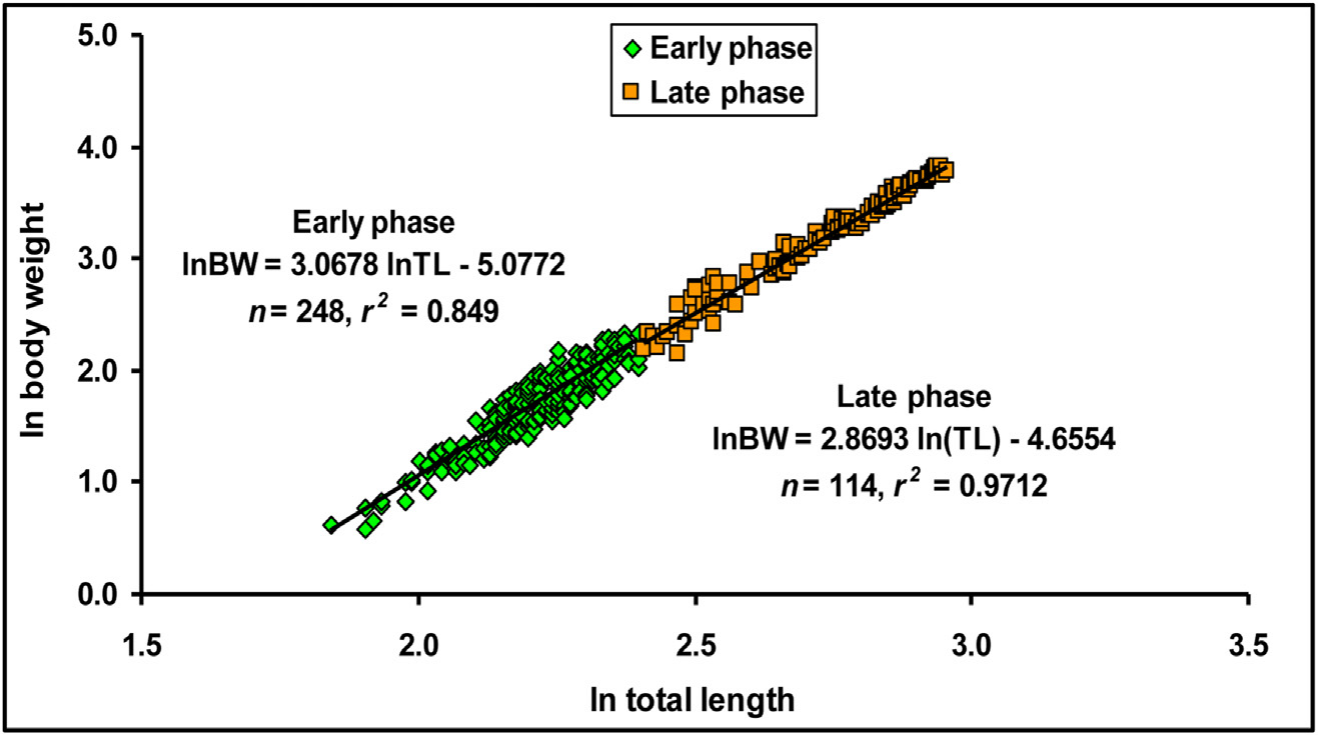
Size at first sexual maturity of *Eutropiichthys vacha* (Hamilton, 1822) in the Ganges River, northwestern Bangladesh.

The estimated *a*_*3.0*_ of males, females, and combined sex of *E. vacha* was 0.0063, 0.0066, and 0.0065 respectively, signifying the elongated body shape in the Ganges River (Table 8). Also, the assessed *L*_*m*_ based on *L*_*max*_ for both sexes was 11.38 cm (95% CL = 9.01–14.38 cm TL) and 11.27 cm TL (95% CL = 8.92–14.24 cm TL), respectively (Figure 6 and Table 8). Besides, *a*_*3.0*_ and *L*_*m*_ were also calculated from various water bodies using available literature are given in Table 9.

**Table 8:** Estimation of form factor, size at first sexual maturity, asymptotic length, asymptotic weight, natural mortality and optimum catchable length of *Eutropiichthys vacha* in the Ganges River using different models based on maximum length and life history parameters.

Sex	Regression parameters		*L*_*max*_ (cm)	*a*_*3.0*_	*L*_*m*_ (95% CL of *L*_*m*_*)*	*L*_*∞*_	*W*_*∞*_	Calculated *M*_*w*_ (year^−1^)				Calculated *L*_*opt*_ (cm)		
	*a*	*b*						Peterson and Wroblewski (1984)	Hoenig (1983)	Jensen (1996)	Mean	Froese and Binohlan (2000)	Beverton (1992)	Mean
M	0.0094	2.87	19.20	0.0063	11.38 (9.01–14.38)	20.27	51.41	1.07	1.454	1.353	1.292	12.24	14.92	13.58
F	0.0099	2.87	19.00	0.0066	11.27 (8.92–14.24)	20.06	54.15	1.06	1.433	1.370	1.288	12.10	14.07	13.09
C	0.0097	2.87	19.20	0.0065	11.38 (9.01–14.38)	20.27	54.65	1.08	1.454	1.353	1.296	12.24	14.08	13.16

M, Male; F, Female; C, Combined; CL, confidence limit; *L*_*max*_, maximum length; *a*_*3.0*_, form factor; *L*_*m,*_ Size at first sexual maturity; *L*_*∞*_, asymptotic length; *W*_*∞*,_ asymptotic weight; *M*_*w*_, Natural mortality; *L*_*opt*_, optimum catchable length.

**Table 9:** The calculated form factor, size at first sexual maturity, asymptotic length, natural mortality and optimum catchable length of *Eutropiichthys vacha* (Hamilton, 1822) from worldwide different water-bodies.

Water body	Sex	Regression parameters		*L*_*max*_	*a*_*3.0*_	*L*_*m*_ (cm)	95% CL of *L*_*m*_	*L*_*∞*_ (cm)	*M*_*w*_ (y^1^)	*L*_*opt*_ (cm)	References
		*a*	*b*								
Padma River, Bangladesh	U	0.1070	2.99	21.30	0.1037	12.52	9.86–15.87	22.45	0.49	13.61	Hossain (2010)
Ganges River, Bangladesh	U	0.0180	2.84	25.80	0.0109	14.92	11.66–19.03	27.11	0.82	16.57	Hossain et al. (2009)
Ganges River, Bangladesh	M	0.0087	2.86	25.80	0.0056	14.92	11.66–19.03	27.11	0.91	16.57	Hossain et al. (2012)
	F	0.0091	2.87	27.00	0.0061	15.55	12.14–19.86	28.35	0.85	17.36	
Jamuna River, Bangladesh	M	0.0060	3.03	16.94	0.0066	10.15	8.07–12.78	17.92	1.04	10.76	Hossain et al. (2013)
	F	0.0090	2.81	16.95	0.0050	10.15	8.07–12.78	17.93	1.08	10.77	
Kaptai Lake, Bangladesh	C	0.0088	2.96	42.5	0.0078	23.56	18.06–30.52	44.40^a^	2.17^a^^,^^b^	27.70	Bashar et al. (2021)
Kaptai Lake, Meghna River and Tanguar *haor*, Bangladesh	U	–	–	28.40	–	16.29	12.69–20.84	29.8	–	18.28	Parvej et al. (2014)
Atrai and Brahmaputra River, Bangladesh	U	0.0110	2.83	18.50	0.0065	11.00	8.72–13.79	19.54	1.06	11.78	Islam et al. (2017)
Ganges River, Bangladesh	M	0.0103	2.83	19.90	0.0061	11.76	9.29–14.88	21.00	1.01	12.70	Khatun et al. (2018)
	F	0.0120	2.78	20.60	0.0060	12.14	9.58–15.37	21.73	1.02	13.16	
Betwa and Gomti River, India	C	0.0138	2.73	21.50	0.0059	12.62	9.94–16.01	22.66	0.93	13.74	Sani et al. (2010)
Ganga River, India	C	–	–	37.00	–	20.75	15.99–26.76	38.66	–	23.98	Tripathi et al. (2015)
Damodor River, India	U	–	–	18.00	–	10.73	8.51–13.53	19.02	–	11.45	Khatun and Chakraborti (2016)
Indus River, Pakistan	M	–	–	31.50	–	17.91	13.89–22.98	33.00	–	20.33	Soomro et al. (2012)
	F	–	–	34.00	–	19.21	14.85–24.71	35.57	–	21.99	
Indus River, Pakistan	M	0.0039	3.16	21.50	0.0064	12.62	9.94–16.01	22.66	0.80	13.74	Soomro et al. (2007)
	F	0.0072	2.96	21.50	0.0064	12.62	9.94–16.01	22.66	0.79	13.74	
Indus River, Pakistan	M	0.0140	2.75	32.00	0.0064	18.17	14.09–23.33	33.6^a^	1.13^a^^,^^c^	20.72	Memon et al. (2017)
	F	0.0170	2.67	34.00	0.0061	19.21	18.45–24.71	35.7^a^	1.04^a^^,^^c^	22.07	
Salween River, China	C	–	–	40.20	–	22.39	12.20–29.95	41.95	–	26.11	Hora et al. (1941)

M, Male; F, Female; C, Combined; U, Unsexed; *a*, intercept; *b*, slope; *L*_*max*_, maximum length; *a_3.0_*, form factor; *L*_*m*_, Size at first sexual maturity; CL, confidence limits; *L*_*∞*_, asymptotic length, *M*_*W*_, Natural mortality (estimated based on Peterson and Wroblewski, 1984), *L*_*opt*_, optimum catchable length (calculated based on Froese and Binohlan, 2000).

a original value provided by the authors.

b mortality calculated based on Jensen (1996)’s equation.

c mortality calculated based on Pauly (1980)’s equation.

### 3.5 Natural mortality (*M*_*W*_)

**Figure 7:**
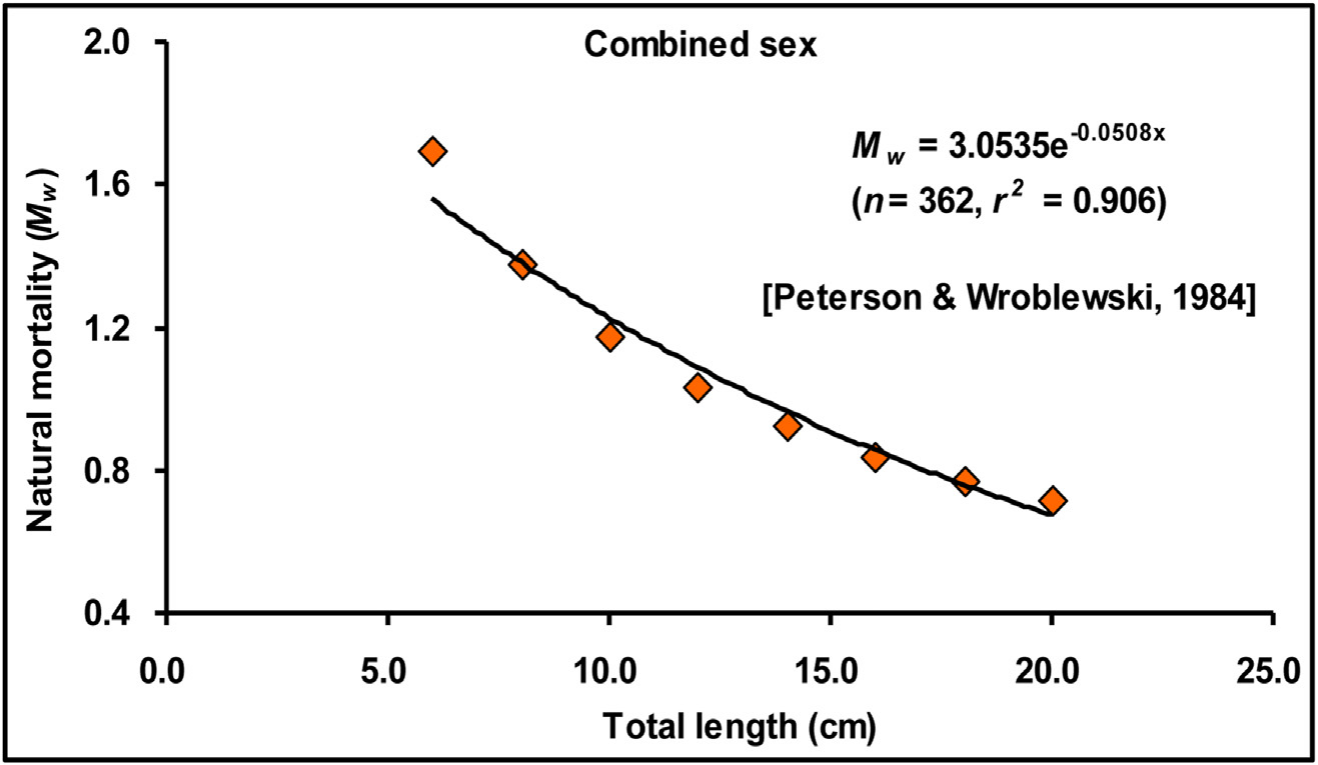
The natural mortality of *Eutropiichthys vacha* (Hamilton, 1822) in the Ganges River, northwestern Bangladesh.

Table 8 displays the calculated natural mortality based on three distinct models along with their mean value. The values of mortality estimated by Hoenig (1983) and Jensen (1996) in this study were comparatively higher than the estimates of the Peterson and Wroblewski (1984) model. However, the mean *M*_*W*_ for the *E. vacha* population was calculated to be 1.29 year^−1^ for males and 1.28 year^−1^ for females (Table 8) in the Ganges River of NW Bangladesh. In addition, Figure 7 represents the relationship between the total length and natural mortality, which indicates that *M*_*W*_ in the Ganges River was quite high for specimens below 6.00 cm TL, but it decreased as body size increased. The calculated *M*_*W*_ for *E. vacha* from different water bodies around the world is shown in Table 9.

### 3.6 Optimal catchable length (*L*_*opt*_)

**Figure 8:**
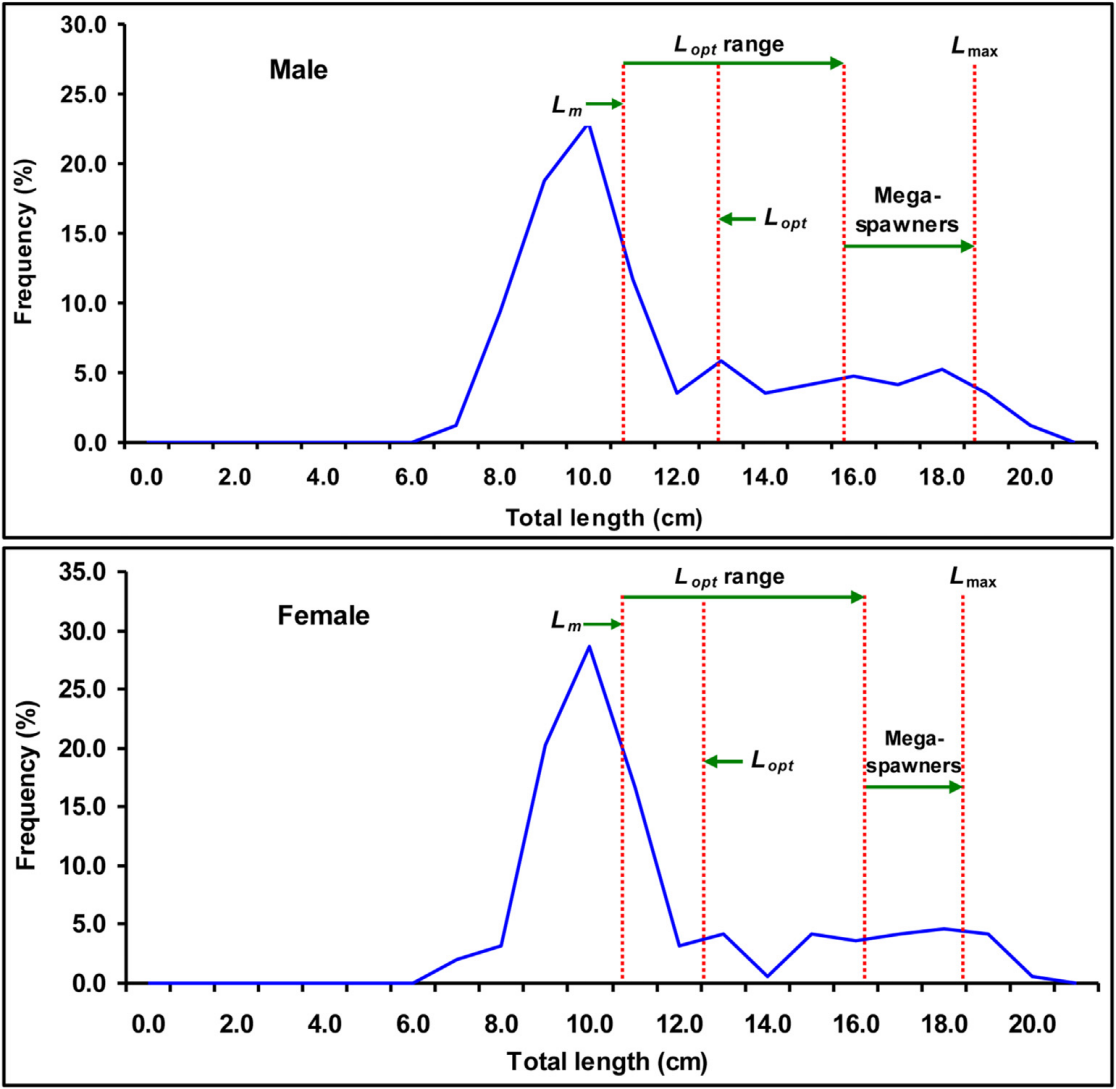
Optimum catchable length of *Eutropiichthys vacha* stock from the Ganges River.

The calculated *L*_*opt*_ by two different models and their mean value are given in Table 8 and Figure 8. Also, the *L*_*∞*_*, L*_*m*_, and *L*_*opt*_ were calculated based on *L*_*max*_ using previous literature reported by several scientists from various water bodies on *E. vacha* is given in Table 9.

## 4 Discussion

Development of conservation strategies for wild fishes in the inland waters greatly relies on the documentation and regular upgradation of life history traits (Chowdhury et al., 2021). As fisheries management is a dependent field, concrete data on demography parameters (i.e., growth, mortality, recruitment, etc.) are vital prerequisites for the implementation of proper management policies (Raghavan et al., 2018; Gosavi et al., 2019). *E. vacha* is currently listed as least concern, but still facing different threats which may lead it to revert to its previous condition (critically endangered) if proper management measures cannot be implemented. Hence, detailed investigation of life history parameters might act as a key solution to manage this species sustainably in its natural habitats. Therefore, this study emphasized the estimation of life history parameters of *E. vacha* comprising SR, LFDs, LWRs, LLRs, condition factors (*K*_*A*_*, K*_*F*_*, K*_*R*_ and *W*_*R*_), *a*_*3.0*_, *L*_*m*_, *M*_*W*_, and *L*_*opt*_ using multi-models from the Ganges River in NW Bangladesh.

The majority of aquatic (fish and shellfish) species are unlikely to depart from a typical male-to-female ratio of 1:1. However, this ratio may be significantly prejudiced in many finfish and prawn populations or even within the same population at different time period (Oliveira et al., 2012; Khatun et al., 2018), depending on a number of variables such as reproductive behavior, population adaptability, food accessibility, and environmental circumstances (Brykov et al., 2008; Vandeputte et al., 2012). During this study, out of 362 studied specimens of *E. vacha*, the male to female sex ratio was 1:1.13, with females outnumbering males. In contrast to males, reproductive success of females is typically influenced by resource availability and environmental factors rather than the quantity of mating partners. Consequently, reproductive success of males throughout the lifespan being restricted by the accessibility to females, which might be a reason of imbalance in the number of individuals of each sex in the studied population (Forsgren et al., 2008). Similar to this study, Khatun et al. (2018) from the Ganges River, Bangladesh (male: female = 1.0:1.13) and Tripathi et al. (2015) from the Ganga River, India (male: female = 1.0:1.61) found female-dominated populations, whereas the male dominance observed in the Indus River population of Pakistan (male: females = 1.16: 1.0) reported by Soomro et al. (2012). The total sex ratio in the current investigation did not deviate significantly from the typical 1:1 ratio (*df* = 1, χ^2^ = 1.34, *p* < 0.05), which is consistent with the finding of Hossain et al. (2013) (*df* = 1, χ^2^ = 2.57, *p* > 0.05) from the Jamuna River, northern Bangladesh.

The examined specimens of this study showed total length and weights ranging between 6.3 to 19.2 cm (Mean = 11.09 ± 3.23) and 1.80–45.65 g (Mean = 11.950 ± 10.923), respectively, throughout the whole year. The length of the largest individuals (*L*_*max*_) known from a population has strong correlation with asymptotic length (*L*_*∞*_) (Froese and Binohlan, 2000). The calculated *L*_*∞*_ of *E. vacha* in this study was 20.27 and 20.06 cm for male and female, respectively (Table 8). Since the *L*_*∞*_ was calculated using empirical formula based on *L*_*max*_, *L*_*∞*_ does not revealed any obvious trend during pre and post assessment of IUCN Bangladesh (2015) assessment for *E. vacha* from Bangladesh (Table 9). The highest *L*_*∞*_ was found for Kaptai Lake population (44.40 cm) reported by Bashar et al. (2021). However, special care are needed for comparison of observed variation of *L*_*∞*_ in this study, because methodology and sampling gear may affect the size ranges of sampled individuals and their resultant size-based parameters (Heino et al., 2011; Kaartvedt et al., 2012). All LWRs in this study exhibited a highly significant correlation (*p* < 0.001) with the *r*^*2*^ values ≥0.975. These high *r*^*2*^ values indicate good accuracy for the prediction of a linear regression for the studied fish species, and suggests that projection in future catches in this geographical area for this size range is possible.

The allometric co-efficient (*b*) can range from 2 to 4 (Carlander, 1969), but values in the 2.5–3.5 range are more common (Froese, 2006). Tesch (1971) stated that *b* values near to 3 specify isometric growth while differences from 3 designate allometric growth, either positive (>3) or negative (<3) allometric. The *b* values of all LWRs (TL *vs.* BW; FL *vs.* BW; SL *vs.* BW) in the current study were 2.85–2.91 for males and 2.87–2.93 for females, suggesting negative allometric growth pattern (<3.00) for both sexes which is indicative of a relatively slow growth rate and thinner bodies. For the female population, similar growth pattern was described by Hossain et al. (2013) (*b* = 2.81), Khatun et al. (2018) (*b* = 2.78) and Soomro et al. (2007) (*b* = 2.96). Khatun et al. (2018) reported a similar growth pattern (*b* = 2.83) in the male population of the Ganges River in Bangladesh. However, contrary to the present findings, Hossain et al. (2013) observed isometric growth (*b* = 3.03), while Soomro et al. (2007) showed positive allometric growth (*b* = 3.16) for male populations of *E. vacha* from the respective Jamuna River of Bangladesh, and the Indus River of Pakistan. While we are unable to provide a definitive explanation for the similarities and differences between our findings and the previous literatures, it is possible that the state of the species, and its geographic differences in population structure can be responsible (Jisr et al., 2018). Moreover, these distinctions may be derived due to several factors, including sex, season, maturity stages, and magnitude of stomach fullness (Ali et al., 2016; Ogunola et al., 2018; Czudaj et al., 2022). Thus, this observed variation might be assumed to be the impact of a single factor or a synergistic effect of multiple factors (Gosavi et al., 2019).

Despite the fact that most studies focused on a specific condition factor, we investigated four condition factors (*K*_*A*_, *K*_*F*_, *K*_*R*_ and *W*_*R*_) to assess the viability and habitat quality of *E. vacha* in the Ganges River. *K*_*R*_ is a crucial condition factor which can reveal the physiological and nutritional status of an individual or a population. It is mostly interpreted with respect to energy reserves as well as with life history characteristics, such as reproduction and growth (Gubiani et al., 2020). Within the same sample or population, Froese (2006) suggested to use *K*_*R*_ for comparison of the health status of males and females. Information on differences in food availability and the impact of physicochemical parameters on the life cycle of fish species is shown by the divergence of *K*_*R*_ from 1, where *K*_*R*_ > 1 implying good general condition and *K*_*R*_ < 1 indicate opposite condition (Le Cren, 1951). The sex-wise analysis of maximum *K*_*R*_ values in males (1.50) was higher than females (0.67) (Table 6), suggesting that males are in better condition in comparison to females in the study area. This disparity could be attributed to the weight of food in the stomachs of the sexes (Ambily and Nandan, 2010). In the case of *K*_*F*_, male fishes likewise displayed higher values than female fishes (Table 6), indicating that overall, the general health of male fishes was better than female fishes. Hossain et al. (2013) reported the same result. This may be due to increased appetite and feeding intensity of male fishes than females (Subba et al., 2018). According to Barnham et al. (2003), the stage of development of reproductive organs has a significant impact on the *K*_*F*_, which in females drops quickly after the eggs are shed. In this study, females attains sexual maturity relatively faster than males (Table 8) which may also be responsible for lower *K*_*F*_ in females. The Spearman rank correlation test revealed that only *K*_*F*_ demonstrated a significant correlation with TL and BW for both sexes, compared to the other condition factors (Table 7). Therefore, this study suggested that *K*_*F*_ could be used to assess the welfare of this fish in the Ganges River and its neighboring ecological community. The mean *W*_*R*_ of both sexes tended to be 100 (Table 6), demonstrating the presence of prey and predator in this waterbody was balanced. However, the Wilcoxon signed rank test revealed that the *W*_*R*_ for males (*p* = 0.845) indicated no noteworthy variations from 100, but a significant difference for females (*p* < 0.0001), signifying that the habitat was optimal for males but not for females, suggesting an imbalance in food accessibility comparative to predator existence (Anderson and Neumann, 1996) for *E. vacha* in the Ganges River watershed area. According to Blackwell et al. (2000) when fish species have fairly limited or well-defined diets, only then *W*_*R*_ may act as a good indicator to predict the availability of prey, and they advised that the association between *W*_*R*_ and prey (i.e., food habits) should be confirmed before drawing such assumption. We did not study the food habits of *E. vacha* and thus addressing its necessity to include in future studies to get a better insight about this sex-specific variation of prey availability in the Ganges River.

The *a*_*3.0*_ assists in determining how significantly an individual's body shape differs from that of others in a particular species or population (Froese, 2006). The calculated *a*_*3.0*_ values of *E. vacha* for both sexes (0.0063 and 0.0066) indicated an elongated body form in the Ganges River. Similar body shapes were also observed for the population of the Jamuna River reported by Hossain et al. (2013) for respective sexes (0.0066 and 0.0055), thus ruling out the likelihood of water body-wise differences of *a*_*3.0*_ within the same country. The calculated *L*_*m*_ of *E. vacha* were 11.38 cm, and 11.27 cm in TL for males and females, accordingly which were smaller than the observed *L*_*m*_ (13.15 for males and 14.00 cm for females) described by Hossain et al. (2012). Khatun et al. (2018) found the *L*_*m*_ to be 12.1 cm TL for females from the Ganges River. Moreover, Sarkar et al. (2017) reported the *L*_*m*_ to be 15.6 cm TL from the upper Ganga Basin, India. The calculated *L*_*m*_ of this study is lower than all these estimates. *L*_*m*_ was estimated based on maximum length in this study, hence it might be a source for this biasness. Moreover, population densities, sample size variation, contraction in the specimen body structure due to formalin preservation (Hossain et al., 2012), temperature of water surface, and availability of foodstuff may all contribute to these differences (Khatun et al., 2019).

In this study, the *M*_*w*_ of *E. vacha* for the Ganges River population was assessed using three different models. Each of these models has its own benefits and limitations, and none of them is a generally accepted model for the calculation of the actual value of natural mortality (Maunder et al., 2011). Therefore, a mean value of *M*_*w*_ was calculated in this study which provides an estimate of *M*_*w*_ as 1.29 year^−1^ for males and 1.28 year^−1^ for females. Bashar et al. (2021) estimated the *M*_*w*_ for combined sexed *E. vacha* as 1.27 year^−1^ from the Kaptai Lake in Bangladesh using the Pauly’s (1980) empirical model, which were not employed in this study. However, the calculated *M*_*w*_ reported by Bashar et al. (2021) following the Jensen (1996) maturity-based model provides a much higher value of 2.17 year^−1^ for combined sex than in this study (Table 8), which might be attributed due to higher *L*_*m*_ of 23.56 cm TL than the observed *L*_*m*_ in this study. However, Kaptai Lake is an artificial lake whose ecological condition is different from the riverine condition. Although using different method (Pauly, 1980), Memon et al. (2017) reported natural mortality as 1.13 and 1.04 year^−1^ for male and female, respectively from the Indus River of Pakistan, however no study have been conducted from any river of Bangladesh to date. Therefore, the estimated value of *M*_*w*_ in this study can be a comparison baseline for future studies for the riverine environment of Bangladesh.

During this study, the *L*_*opt*_ was also estimated using two different models as they may differ in their accuracy. Moreover, their suitability might be in question when used singly (Mawa et al., 2021). To avoid this problem, the mean value of *L*_*opt*_ was calculated and used in this study. According to FAO responsible fisheries strategy, *L*_*opt*_ must be greater than the mean *L*_*m*_ to protect the abundance of stock, allowing a significant proportion of the stock to get spawning opportunities before being captured (Achmad et al., 2020). *L*_*opt*_ also assist in the selection of appropriate mesh size of fishing gear to prevent the capture of fish species below this catchable size as the target capture. The calculated mean *L*_*opt*_ for both sexes in this study was higher than the *L*_*m*_ (Figure 8), indicating the fishing gear is still in favor of reproductive potential for *E. vacha* in the Ganges River. However, small percentage of mega-spawners represents the persistence of recruitment overfishing, hence this study strongly recommend protecting the highest number of brood fish belonging to mega-spawners group in order to ensure the long-term sustainability of this fish every year in the Ganges River.

## 5 Conclusion and recommendations

In the Ganges River, multi-species fisheries are usually practiced with a substantial quantity of by-catch, thus suggesting management measures for single species are fairly cumbersome. Sex-specific analysis of condition factors in this study shows quite unfavorable environment for *E. vacha* in the Ganges River. The calculated *L*_*m*_ was lower than Lopt, hence recommending increased mesh size of fishing gear would be forthright, which will be useful to reduce fishing mortality, but the exploitation rate will be decreased as well which lead to financial losses to the fishermen. Therefore, this study suggests to capture fishes over *L*_*opt*_ so that most of the individual get a chance to reproduce before being caught. This will reduce growth and recruitment overfishing and will ensure higher catches in the long run. However, this study emphasizes the need for a complete study about mortality and exploitation status of *E. vacha* to get a better insight into the current stock condition in the Ganges River. We hope the outcomes of this study will be a functioning tool for fishery managers to initiate appropriate management approaches and regulations for the sustainable conservation of the lingering stocks of this species in the Ganges River and adjacent ecosystem.

## Declarations

### Author contribution statement

Dalia Khatun and Md. Yeamin Hossain: Conceived and designed the experiments; Performed the experiments; Analyzed and interpreted the data; Contributed reagents, materials, analysis tools or data; Wrote the paper.

Obaidur Rahman: Performed the experiments; Analyzed and interpreted the data.

Md. Firose Hossain: Analyzed and interpreted the data; Contributed reagents, materials, analysis tools or data.

### Funding statement

This research did not receive any specific grant from funding agencies in the public, commercial, or not-for-profit sectors.

### Data availability statement

Data will be made available on request.

### Declaration of interest’s statement

The authors declare no conflict of interest.

### Additional information

No additional information is available for this paper.

## References

[bib1] Abbas A. (2010). Food and feeding habits of freshwater catfish *Eutropiichthys vacha* (Bleeker). Indian J. Sci. Res..

[bib2] Achmad D.S., Sudirman, Jompa J., Nurdin M.S. (2020). Estimating the catchable size of orange-spotted grouper (*Epinephelus coioides*) in kwandang bay, gorontalo utara district, Indonesia. Earth Environ. Sci..

[bib3] Ali R.A.S., Elawad A.N., Khalifa M.M., El-Mor M. (2016). Length–weight relationship and condition factor of *Liza ramada* from Eastern coast of Libya. Int. J. Fish. Aqua. Res..

[bib4] Ambily V., Nandan S.B. (2010). Length-weight relationship, relative condition factor (Kn) and morphometry of *Arius subrostratus* (Valenciennes, 1840) from a coastal wetland in Kerala. Indian J. Fish..

[bib5] Anderson R.O., Neumann R.M., Murphy B.R., Willis W.D. (1996). Fisheries Techniques.

[bib7] Azad M.A.K., Hossain M.Y., Khatun D., Parvin M.F., Nawer F., Rahman O., Hossen M.A. (2018). Morphometric relationships of the tank goby *Glossogobius giuris* (Hamilton, 1822) in the Gorai River using multi-linear dimensions. Jordan J. Biol. Sci..

[bib8] Azadi M.A., Islam M.A., Solaiman S. (1990). Some aspects of reproductive biology of *Eutropiichthys vacha* (ham.) in Kaptai Lake, Bangladesh. Chittagong Univ. Stud. Part II Sci..

[bib9] Baitha R., Sinha A., Koushlesh S.K., Chanu T.N., Kumari K., Gogoi P., Ramteke M.H., Borah S., Das B.K. (2018). Length–weight relationship of ten indigenous freshwater fish species from Gandak River, Bihar, India. J. Appl. Ichthyol..

[bib116] Barnham A.C., Baxter F.A., Victoria (2003). Condition factor, K, for Salmonid fish.

[bib13] Bashar M.A., Rahman M.A., Uddin K.B., Ahmed F.F., Mahmud Y., Hossain M.Y. (2021). Assessing the exploitation status of main catfish *Eutropiichthys vacha* based on length based stock assessment models in the Kaptai Lake from Bangladesh. Heliyon.

[bib14] Beverton R.J.H. (1992). Patterns of reproductive strategy parameters in some marine teleost fishes. J. Fish. Biol..

[bib15] Binohlan C., Froese R. (2009). Empirical equations for estimating maximum length from length at first maturity. J. Appl. Ichthyol..

[bib16] Blackwell B.G., Brown M.L., Willis D.W. (2000). Relative weight (Wr) status and currentuse in fisheries assessment and management. Rev. Fish. Sci..

[bib17] Brodziak J., Ianelli J., Lorenzen K., Methot R.D. (2011).

[bib18] Brykov V.A., Kukhlevskiĭ A.D., Shevliakov E.A., Kinas N.M., Zavarina L.O. (2008). Sex ratio control in pink salmon (*Oncorhynchus gorbuscha*) and chum salmon (*O. keta*) populations: the possible causes and mechanisms of changes in the sex ratio. Genetika.

[bib19] Burnham K.P., Anderson D.R. (2002).

[bib20] Carlander K.D. (1969).

[bib21] Chowdhury M.M., Siddiqui K.U., Islam M.A., Kabir S.M.H., Ahmed M., Ahmed A.T.A., Rahman A.K.A., Haque E.U., Ahmed Z.U., Begum Z.N.T., Hassan M.A., Khondker M., Rahman M.M. (2007). Encyclopedia of flora and fauna of Bangladesh.

[bib22] Chowdhury A.A., Hossain M.Y., Mawa Z., Khatun D., Islam M.A., Rahman M.A., Hasan M.R., Rahman O., Konok R.H., Rahman M.A., Parvin M.F., Sarkar U.K. (2021). Some biological aspects of the spotted snakehead *Channa punctata* (Bloch, 1793) in the wetland ecosystem, Gajner Beel, North-western Bangladesh. Indian J. Fish..

[bib23] Christensen V., Walters C. (2004). Ecopath with ecosim: methods, capabilities and limitations. Ecol. Model..

[bib24] Craig J.F., Halls A.S., Barr J.J.F., Bean C.W. (2004). The Bangladesh floodplain fisheries. Fish. Res..

[bib25] Czudaj S., Möllmann C., Fock H.O. (2022). Length–weight relationships of 55 mesopelagic fishes from the eastern tropical North Atlantic: across- and within-species variation (body shape, growth stanza, condition factor). J. Fish. Biol..

[bib26] Das A.K., Manna R.K., Rao D.S.K., Jha B.C., Naskar M., Sharma A.P. (2017). Status of the River Krishna: water quality and riverine environment in relation to fisheries. Aquat. Ecosys. Health Manag..

[bib27] Das I., Hazra S., Das S., Giri S., Maity S., Ghosh S. (2019). Present status of the sustainable fishing limits for Hilsa Shad in the northern Bay of Bengal, India. Proc. Natl. Acad. Sci. India B Biol. Sci..

[bib28] Eschmeyer W.N., Fong J. (2014). Species by Family/Subfamily in the Catalog of Fishes. Catalog of Fishes.

[bib30] Forsgren E., Reynolds J.D., Berglund A., Mooi R.D. (2008). Behavioural ecology of reproduction in fish. Handbook of Fish Biology and Fisheries.

[bib31] Froese R. (2004). Keep it simple: three indicators to deal with overfishing. Fish Fish..

[bib32] Froese R. (2006). Cube law, condition factor and weight length relationships: history, meta-analysis and recommendations. J. Appl. Ichthyol..

[bib33] Froese R., Binohlan C. (2000). Empirical relationships to estimate asymptotic length, length at first maturity and length at maximum yield per recruit in fishes, with a simple method to evaluate length frequency data. J. Fish. Biol..

[bib34] Froese R., Pauly D. (2021). Fishbase, World wide web electronic publication version 2/2021.

[bib35] Fulton T.W. (1904).

[bib36] Garcia C.B., Duarte J.O., Sandoval N., Schiller D., Melo G., Navajas P. (1998). Length-weight relationships of demersal fishes from the gulf of salamanca, Colombia. NAGA. ICLARM Q.

[bib37] Gosavi S.M., Kharat S.S., Kumkar P., Tapkir S.D. (2019). Assessing the sustainability of lepidophagous catfish, *Pachypterus khavalchor* (Kulkarni, 1952), from a tropical river Panchaganga, Maharashtra, India. J. Basic Appl. Zool..

[bib38] Grande L., Eastman J.T. (1986). A review of Antarctic ichthyofaunas in the light of new fossil discoveries. Palaeontology.

[bib39] Gubiani É.A., Ruaro R., Ribeiro V.R., de Santa Fé Ú.M.G. (2020). Relative condition factor: Le Cren’s legacy for fisheries science. Acta Limnol. Bras..

[bib113] Hamilton F. (1822). An account of the fishes found in the river Ganges and its branches. Archibald Constable 1.

[bib40] Hasan M.F., Molla A.H., Ahsan M.S., Alam M.T. (2002). Physicochemical properties and fatty acids distribution pattern in lipids of *Eutropiichthys vacha* Hamilton-Buchanan (Family Schilbeidae). Pakistan J. Biol. Sci..

[bib117] Heino M., Porteiro F.M., Sutton T.T., Falkenhaug T., Godø O.R., Piatkowski U. (2011). Catchability of pelagic trawls for sampling deep-living nekton in the mid-North Atlantic. ICES J. Mar. Sci..

[bib41] Hoenig J.M. (1983). Empirical use of longevity data to estimate total mortality rates. Fish. Bull..

[bib42] Hora S.L. (1941). Siluroid fishes of India, Burma and ceylon. XI. Fishes of the schilbeid genera *silonopangasius* hora, *pseudeutropius* bleaker, *proeutropiichthys* hora and *ailia* gray. Rec. Indian Mus. (Calcutta).

[bib43] Hossain M.Y. (2010). Length-Weight, Length-length relationships and condition factor of three schilbid catfishes from the Padma River, northwestern Bangladesh. Asian Fish Sci..

[bib44] Hossain M.Y., Jasmine S., Ibrahim A.H.M., Ahmed Z.F., Rahman M.M., Ohtomi J. (2009). Length-weight and length-length relationships of 10 small fish species from the Ganges, Bangladesh. J. Appl. Ichthyol..

[bib45] Hossain M.Y., Jewel M.A.S., Nahar L., Rahman M.M., Naif A., Ohtomi J. (2012). Gonadosomatic index-based size at first sexual maturity of the catfish *Eutropiichthys vacha* (Hamilton 1822) in the Ganges River (NW Bangladesh). J. Appl. Ichthyol..

[bib46] Hossain M.Y., Rahman M.M., Jewel M.A.S., Hossain M.A., Ahamed F., Tumpa A.S., Abdallah E.M., Ohtomi J. (2013). Life history traits of the critically endangered catfish *Eutropiichthys vacha* (Hamilton 1822) in the Jamuna (Brahmaputra River distributary) River, northern Bangladesh. Sains Malays..

[bib47] Hossain M.Y., Hossen M.A., Mawa Z., Rahman M.A., Hasan M.R., Islam M.A., Khatun D., Rahman M.A., Tanjin S., Sarmin M.S., Bashar M.A., Ohtomi J. (2021). Life-history traits of three ambassid fishes (*Chanda nama, Parambassis lala* and *Parambassis ranga*) from the Mathabhanga River, southwestern Bangladesh. Lakes Reserv..

[bib50] Islam M.R., Azom M.G., Faridullah M., Mamun M. (2017). Length-weight relationship and condition factor of 13 fish species collected from the Atrai and Brahmaputra Rivers, Bangladesh. J. Biodivers. Environ. Sci. (JBES).

[bib10] IUCN Bangladesh (2000). Mahmud-ul-Ameen, Md. Anwarul Islam, Ainun Nishat.

[bib11] IUCN Bangladesh (2015).

[bib51] Jensen A.L. (1996). Beverton and Holt life history invariants result from optimal tradeoff of reproduction and survival. Can. J. Fish. Aquat. Sci..

[bib52] Jin Y., Liu S., Yuan Z., Yang Y., Tan S., Liu Z. (2016). Catfish genomic studies: progress and perspectives. Genomics in Aquaculture. Academic Press.

[bib114] Jisr N., Younes G., Sukhn C., El-Dakdouki M.H. (2018). Length-weight relationships and relative condition factor of fish inhabiting the marine area of the Eastern Mediterranean city, Tripoli-Lebanon. Egypt J. Aquat. Res..

[bib53] Jones T., Phillips B., Williams C.E., Pittock J. (2003).

[bib118] Kaartvedt S., Staby A., Aksnes D.L. (2012). Efficient trawl avoidance by mesopelagic fishes causes large underestimation of their biomass. Mar. Ecol. Prog. Ser..

[bib54] Kar D., Laskar B.A., Nath D. (2006). Fecundity of *Eutropiichthys vacha* Hamilton-Buchanan: a commercially important fish in Assam. Environ. Ecol..

[bib55] Katsanevakis S. (2006). Modelling fish growth: model selection, multi-model inference and model selection uncertainty. Fish. Res..

[bib56] Katsanevakis S. (2014). Multi-model inference and model selection in Mexican fisheries. Ciencia Pesquera.

[bib57] Khatun R., Chakrabarti P. (2016). Histological and surface ultrastructural observations on the saccus vasculosus of *Eutropiichthys vacha* (Hamilton). Int. J. Fish. Aquat. Stud..

[bib58] Khatun D., Hossain M.Y., Parvin M.F., Ohtomi J. (2018). Temporal variation of sex ratio, growth pattern and physiological status of *Eutropiichthys vacha* (Schilbeidae) in the Ganges River, NW Bangladesh. Zool. Ecol..

[bib59] Khatun D., Hossain M.Y., Nawer F., Mostafa A.A., Al-Askar A.A. (2019). Reproduction of *Eutropiichthys vacha* (Schilbeidae) in the Ganges River (NW Bangladesh) with special reference to potential influence of climate variability. Environ. Sci. Pollut. Res..

[bib119] Khatun D., Hossain M.Y., Hossain M.F., Mawa Z., Rahman M.A., Hasan M.R., Islam M.A., Rahman M.A., Hassan H.U., Sikha S.N. (2022). Population parameters of a freshwater clupeid, *Corica soborna* (Hamilton, 1822) from the Ganges River, northwestern Bangladesh. Pakistan J. Zool..

[bib60] Khatun D., Hossain M.Y., Hossen M.A., Rahman O., Hossain M.F., Islam M.A., Rahman M.A., Mawa Z., Hasan M.R., Rahman M.A., Vadas R.L. (2020). Temporal variation of condition and prey-predator status for a schilbid catfish *Eutropiichthys vach*a (Hamilton, 1822) in the Ganges River, northwestern Bangladesh through multi-model inferences. Indian J. Geo-Mar. Sci..

[bib61] King M. (2007).

[bib62] Kostori F.A., Parween S., Islam M.N. (2011). Availability of small indigenous species (SIS) of fish in the Chalan Beel the largest wetland of Bangladesh. Univ. J. Zool., Rajshahi Univ..

[bib63] Kumar R.S., Sarkar U.K., Gusain O., Dubey V.K., Pandey A., Lakra W.S. (2014). Age, growth, population structure and reproductive potential of a vulnerable freshwater mullet, *Rhinomugil corsula* (Hamilton, 1822) from a Tropical River Betwa in Central India. Proc. Natl. Acad. Sci. India B Biol. Sci..

[bib64] Le Cren E.D. (1951). The length-weight relationship and seasonal cycle in gonad weight and condition in the perch (*Perca fluviatilis*). J. Anim. Ecol..

[bib66] Maunder M.N., Wong R.A. (2011). Approaches for estimating natural mortality: application to summer flounder (*Paralichthys dentatus*) in the U.S. mid-Atlantic. Fish. Res..

[bib67] Mawa Z., Hossain M.Y., Hasan M.R., Tanjin S., Rahman M.A., Sarmin M.S., Habib K.A. (2021). First record on size at sexual maturity and optimum catchable length of 10 marine fishes from the Bay of Bengal (Bangladesh) through multi-models approach: a key for sound fisheries management. Environ. Sci. Pollut. Res..

[bib68] Memon A.M., Liu Q., Baloch W.A., Soomro A.N., Mohsin M., Noman M., Karim E. (2017). Population parameters of siluroid catfish (*Eutropiichthys vacha*) from Indus River, Pakistan. Int. J. Agric. Biol..

[bib69] Menon A.G.K. (1999). Checklist - freshwater fishes of India. Rec. Zool. Surv. India. Misc. Publ., Occas. Pap..

[bib70] Muchlisin Z.A., Musman M., Azizah M.N.S. (2010). Length-weight relationships and condition factors of two threatened fishes, *Rasbora tawarensis* and *Poropuntius tawarensis*, endemic to Lake Laut Tawar, Aceh Province, Indonesia.. J. Appl. Ichthyol..

[bib71] Neuman R.M., Allen M.S. (2001).

[bib72] Ogunola O.S., Onada O.A., Falaye A.E. (2018). Preliminary evaluation of some aspects of the ecology (growth pattern, condition factor and reproductive biology) of African pike, *Hepsetus odoe* (Bloch 1794), in Lake Eleiyele, Ibadan, Nigeria. Fish. Aquat. Sci..

[bib73] Oliveira M.R., Costa E.F.S., Araújo A.S., Pessoa E.K.R., Carvalho M.M., Cavalcante L.F.M., Chellappa S. (2012). Sex ratio and length-weight relationship for five marine fish species from Brazil. J. Mar. Biol. Oceanogr..

[bib75] Parvej M.R., Islam M.R., Minar M.H., Hossain M.B., Tushar M.R. (2014). Landmark-based morphometric and meristic variations of the critically endangered catfish, *Eutropiichthys vacha* from three different populations in Bangladesh. World J. Fish Mar. Sci..

[bib76] Pauly D. (1980). On the interrelationships between natural mortality, growth parameters, and mean environmental temperature in 175 stocks. ICES J. Mar. Sci..

[bib78] Pauly D., Munro J.L. (1984). Once more on the comparison of growth in fish and invertebrates. ICLARM: International Center for Living Aquatic Resources Management. Fishbyte.

[bib79] Pervaiz K., Iqbal Z., Mirza M.R. (2012). Length-weight, length-length relationships and feeding habits of wild Indus Mahseer, *Tor macrolepis*, from Attock. Pakistan. J. Appl. Ichthyol..

[bib80] Peterson I., Wroblewski J.S. (1984). Mortality rates of fishes in the pelagic ecosystem. Can. J. Fish. Aquat. Sci..

[bib81] Prasad G., Ali A., Harikrishnan M., Raghavan R. (2012). Population dynamics of an endemic and threatened yellow catfish, *Horabagrus brachysoma* (Gűnther) from River Periyar, Kerala, India. J. Threat. Taxa.

[bib82] Qasim S.Z., Qayyum A. (1961). Spawning frequencies and breeding season of some fresh water fishes with special reference to those occurring in the plains of Northern India. Indian J. Fish..

[bib83] Raghavan R., Ali A., Philip S., Dahanukar N. (2018). Effect of unmanaged harvests for the aquarium trade on the population status and dynamics of redline torpedo barb: a threatened aquatic flagship. Aquat. Conserv. Mar. Freshw. Ecosyst..

[bib84] Rahman A.K.A. (2005).

[bib85] Rahman M.M., Hossain M.Y., Ahamed F., Fatematuzzhura, Subba B.R., Abdallah E.M., OhtomI J. (2012). Biodiversity in the Padma distributary of the Ganges River, northwestern Bangladesh: recommendations for conservation. World J. Zool..

[bib87] Ranjan J.B., Herwig W., Subodh S., Michael S. (2005). Study of the length frequency distribution of sucker head, *Garra gotyla gotyla* (Gray, 1830) in different rivers and seasons in Nepal and its applications. Kathmandu Univ. J. Sci. Eng. Technol..

[bib88] Renjithkumar C.R., Roshni K., Ranjeet K. (2021). Length–weight relations of 14 fish species (actinopterygii) from the Chalakudy River,VWestern Ghats, India. Acta Ichthyol. Piscat..

[bib89] Riede K. (2004). Final Report of the R&D-Project 808 05 081.

[bib91] Sabbir W., Hossain M.Y., Rahman M.A., Hasan M.R., Mawa Z., Tanjin S., Ohtomi J. (2021). First report on reproductive features of the Hooghly croaker *Panna heterolepis* Trewavas, 1977 from the Bay of Bengal in relation to environmental factors. Environ. Sci. Pollut. Res..

[bib92] Sani R., Gupta B.K., Sarkar U.K., Pandey A., Dubey V.K., Lakra W.S. (2010). Length-weight relationships of 14 Indian freshwater fish species from the Betwa (YamunaRiver tributary) and Gomti (Ganga River tributary) rivers. J. Appl. Ichthyol..

[bib93] Sarkar U.K., Naskar M., Roy K., Sudheesan D., Srivastava P.K., Gupta S., Bose A.K. (2017). Benchmarking pre-spawning fitness, climate preferendum of some catfishes from river Ganga and its proposed utility in climate research. Environ. Monit. Assess..

[bib94] Sarker M.Y., Ayub F., Mian S., Lucky N.S., Hossain M.A.R. (2008). Biodiversity status of freshwater catfishes in some selected waterbodies of Bangladesh. J. Taxon. Biodiv. Res..

[bib96] Siddiqui K.U., Islam M.A., Kabir S.M.H., Ahmad M., Ahmed A.T.A., Rahman A.K.A., Haque E.U., Ahmed Z.U., Begum Z.N.T., Hasan A., Khondker M., Rahman M.M. (2007).

[bib97] Sokal R.R., Rohlf F.J. (1987).

[bib98] Soomro A.N., Baloch W.A., Jafri S.I.H., Suzuki H. (2007). Studies on length-weight and length-length relationship of catfish *Eutropiichthyes vacha* Hamilton (Schilbeidae- Siluriformes) from Indus River, sindh, Pakistan. Caspian J. Env. Sci..

[bib99] Soomro A.N., Baloch W.A., Jafri S.I.H., Burdi G.H., Fulanda B. (2012). Reproduction and feeding habits of the river catfish *Eutropiichthys vacha* (Hamilton, 1822) (Siluriformes, schilbidae) in an impacted habitat: kotri hydrodam, river Indus, Pakistan. Our Nat..

[bib100] Subba S., Subba B.R., Mahaseth V.K. (2018). Relative condition factor, length-weight relationship and sex ratio of copper mahseer, *Neolissochilus hexagonolepis* (McClelland, 1839) from Tamor River, Nepal. Our Nat..

[bib101] Talwar P.K., Jhingran A.G. (1991).

[bib102] Templeman W. (1987). Differences in sexual maturity and related characteristics between populations of thorny skate (*Raja radiate*) from the northwest Atlantic. J. Northwest Atl. Fish. Sci..

[bib103] Tesch F.W., Ricker W.E. (1968). Methods for Assessment of Fish Production in Fresh Waters.

[bib104] Tesch F.W., Ricker W.E. (1971). Methods for Assessment of Fish Production in Freshwaters.

[bib105] Tripathi S., Gopesh A., Joshi K.D., Dwivedi A.C. (2015). Size composition, exploitation pattern, sex ratio and sex structure of *Eutropiichthys vacha* (Hamilton, 1822) from the middle stretch of the river Ganga at Allahabad, India. Adv. Biosci. Technol..

[bib106] Vandeputte M., Quillet E., Chatain B. (2012). Are sex ratios in wild Europeansea bass (*Dicentrarchus labrax*) populations biased?. Aquat. Living Resour..

[bib107] Vazzoler A.E.A.M. (1996).

[bib108] Wang L.J., You F., Wang Q.X., Wul Z.H., Liu M.X. (2015). Length-weight and length-length relationships of 11 fish species from Zhimai River estuary, China. J. Appl. Ichthyol..

[bib109] Wilson D.E., Reeder D.M. (2005).

